# SLAM Project - Long Term Ecological Study of the Impacts of Climate Change in the natural forests of Azores: V - New records of terrestrial arthropods after ten years of SLAM sampling

**DOI:** 10.3897/BDJ.10.e97952

**Published:** 2022-12-14

**Authors:** Sébastien Lhoumeau, Pedro Cardoso, Mário Boieiro, Alejandra Ros-Prieto, Ricardo Costa, Lucas Lamelas-Lopez, Abrão Leite, Isabel Amorim do Rosário, Rosalina Gabriel, Jagoba Malumbres-Olarte, François Rigal, Ana M. C. Santos, Noelline Tsafack, Maria Teresa Ferreira, Paulo A. V. Borges

**Affiliations:** 1 cE3c- Centre for Ecology, Evolution and Environmental Changes, Azorean Biodiversity Group, CHANGE – Global Change and Sustainability Institute, Faculty of Agricultural Sciences and Environment, University of the Azores, Rua Capitão João d´Ávila, Pico da Urze, 9700-042, Angra do Heroísmo, Azores, Portugal cE3c- Centre for Ecology, Evolution and Environmental Changes, Azorean Biodiversity Group, CHANGE – Global Change and Sustainability Institute, Faculty of Agricultural Sciences and Environment, University of the Azores, Rua Capitão João d´Ávila, Pico da Urze, 9700-042 Angra do Heroísmo, Azores Portugal; 2 LIBRe – Laboratory for Integrative Biodiversity Research, Finnish Museum of Natural History, University of Helsinki, P.O.Box 17 (Pohjoinen Rautatiekatu 13), 00014, Helsinki, Finland LIBRe – Laboratory for Integrative Biodiversity Research, Finnish Museum of Natural History, University of Helsinki, P.O.Box 17 (Pohjoinen Rautatiekatu 13), 00014 Helsinki Finland; 3 IUCN SSC Mid-Atlantic Islands Invertebrates Specialist Group, Angra do Heroísmo, Azores, Portugal IUCN SSC Mid-Atlantic Islands Invertebrates Specialist Group Angra do Heroísmo, Azores Portugal; 4 Institut Des Sciences Analytiques et de Physico Chimie pour L’environnement et les Materiaux UMR5254, Comité National de la Recherche Scientifique - University de Pau et des Pays de l’Adour - E2S UPPA, Pau Cedex 64013, France Institut Des Sciences Analytiques et de Physico Chimie pour L’environnement et les Materiaux UMR5254, Comité National de la Recherche Scientifique - University de Pau et des Pays de l’Adour - E2S UPPA Pau Cedex 64013 France; 5 Terrestrial Ecology Group (TEG-UAM), Departamento de Ecología, Universidad Autónoma de Madrid, 28049, Madrid, Portugal Terrestrial Ecology Group (TEG-UAM), Departamento de Ecología, Universidad Autónoma de Madrid, 28049 Madrid Portugal; 6 Centro de Investigación en Biodiversidad y Cambio Global (CIBC-UAM), Universidad Autónoma de Madrid, 28049, Madrid, Portugal Centro de Investigación en Biodiversidad y Cambio Global (CIBC-UAM), Universidad Autónoma de Madrid, 28049 Madrid Portugal; 7 Regional Secretariat of Environment and Climate Change, Project LIFE BEETLES (LIFE 18NAT/PT/000864), Rua do Galo n118, 9700-040, Angra do Heroísmo, Azores, Portugal Regional Secretariat of Environment and Climate Change, Project LIFE BEETLES (LIFE 18NAT/PT/000864), Rua do Galo n118, 9700-040 Angra do Heroísmo, Azores Portugal

**Keywords:** Arthropoda, Azores, new records, long-term monitoring, native forests, SLAM trap

## Abstract

**Background:**

A long-term study monitoring arthropods (Arthropoda) is being conducted since 2012 in the forests of Azorean Islands. Named "SLAM - Long Term Ecological Study of the Impacts of Climate Change in the natural forest of Azores", this project aims to understand the impact of biodiversity erosion drivers in the distribution, abundance and diversity of Azorean arthropods. The current dataset represents arthropods that have been recorded using a total of 42 passive SLAM traps (Sea, Land and Air Malaise) deployed in native, mixed and exotic forest fragments in seven Azorean Islands (Flores, Faial, Pico, Graciosa, Terceira, São Miguel and Santa Maria). This manuscript is the fifth data-paper contribution, based on data from this long-term monitoring project.

**New information:**

We targeted taxa for species identification belonging to Arachnida (excluding Acari), Chilopoda, Diplopoda, Hexapoda (excluding Collembola, Lepidoptera, Diptera and Hymenoptera (but including only Formicidae)). Specimens were sampled over seven Azorean Islands during the 2012-2021 period. Spiders (Araneae) data from Pico and Terceira Islands are not included since they have been already published elsewhere (Costa and Borges 2021, Lhoumeau et al. 2022). We collected a total of 176007 specimens, of which 168565 (95.7%) were identified to the species or subspecies level. For Araneae and some Hemiptera species, juveniles are also included in this paper, since the low diversity in the Azores allows a relatively precise species-level identification of this life-stage. We recorded a total of 316 named species and subspecies, belonging to 25 orders, 106 families and 260 genera. The ten most abundant species were mostly endemic or native non-endemic (one Opiliones, one Archaeognatha and seven Hemiptera) and only one exotic species, the Julida *Ommatoiulusmoreleti* (Lucas, 1860). These ten species represent 107330 individuals (60%) of all sampled specimens and can be considered as the dominant species in the Azorean native forests for the target studied taxa. The Hemiptera were the most abundant taxa, with 90127 (50.4%) specimens. The Coleoptera were the most diverse with 30 (28.6%) families.

We registered 72 new records for many of the islands (two for Flores, eight for Faial, 24 for Graciosa, 23 for Pico, eight for Terceira, three for São Miguel and four for Santa Maria). These records represent 58 species. None of them is new to the Azores Archipelago. Most of the new records are introduced species, all still with low abundance on the studied islands. This publication contributes to increasing the baseline information for future long-term comparisons of the arthropods of the studied sites and the knowledge of the arthropod fauna of the native forests of the Azores, in terms of species abundance, distribution and diversity throughout seasons and years.

## Introduction

A common finding all over the globe is that arthropods are the major taxa involved in ecosystems services (Weisser and Siemann 2013, Gullan and Cranston 2014, Jones et al. 2014, Jankielsohn 2018). Some of these services are now well studied, such as pollination (Pey et al. 2014, Cross et al. 2015) or biological control, food provisioning and recycling organic matter (Noriega et al. 2018). Nevertheless, to fully understand how these taxa shape human activities, the first step is to survey their diversity, abundance structure and variation through time (Dornelas et al. 2013). Although the arthropod fauna is one of the most diverse taxon on Earth (Gullan and Cranston 2014, Aberlenc 2020), its diversity is still poorly documented (Sallé et al. 2021, Bukowski et al. 2022).

Islands are critical places for the conservation of biodiversity, there being a critical need to gather knowledge to support conservation management in such extremely dynamic and changing ecosystems (Whittaker and Fernández-Palacios 2007, Fernández-Palacios et al. 2021, Wyckhuys et al. 2022). They harbour a unique diversity with often high levels of endemicity amongst many taxa (MacArthur and Wilson 2001, Borges et al. 2019, Fernández-Palacios et al. 2021, Florencio et al. 2021). However, as a consequence of their particular evolutionary process, island communities are also very sensitive to the introduction of exotic species (Sax and Gaines 2008, Pyšek et al. 2020). These new arrivals are more and more frequent due to the increase in the transit of people and goods, which offers new opportunities for species to spread rapidly over large areas (Jenkins 1996, Nentwig 2008, Kueffer et al. 2010). Therefore, monitoring and documenting changes in arthropod communities are urgent, especially on islands, to guide and improve biodiversity management strategies (Loh et al. 2005, Blüthgen et al. 2022). Standardised tools and protocols offer the possibility to reproduce science and more defined results on species distributions and their dynamics (Carvalho et al. 2012, Borges et al. 2018, Malumbres-Olarte et al. 2019). Furthermore, importance must be given to the new records as they appear to be new pieces in the complex puzzle of life (Borges and Wunderlich 2008, Borges et al. 2013, Bolu and Varga 2021, Borges et al. 2022a), especially on islands where they are more likely to be introduced species that can threaten the sustainability of ecosystems (Tylianakis et al. 2008, Albrecht et al. 2014, Heleno et al. 2020).

## General description

### Purpose

This publication provides an inventory of terrestrial arthropods present in mixed and native forests of seven Azorean Islands (Flores, Faial, Graciosa, Pico, Terceira, São Miguel and Santa Maria). This is the fifth data paper contribution to the long-term project SLAM (Long Term Ecological Study of the Impacts of Climate Change in the natural forest of Azores) that started in 2012 with the aim of understanding the impact of the drivers of biodiversity erosion on Azorean native forests (Azores, Portugal) (see previous data papers in Costa and Borges (2021), Borges et al. (2022c), Borges et al. (2022d), Lhoumeau et al. (2022)).

This long-term project aims to (Costa and Borges 2021):

1. collect long-term ecological data to evaluate species distributions and abundance at multiple spatial and temporal scales, responding to the Wallacean and Prestonian shortfalls (Cardoso et al. 2011a);
2. identify biodiversity erosion drivers impacting oceanic indigenous assemblages under global change for conservation management purposes;
3. investigate species-environment relationships and use species distribution and abundance data in model-based studies of environmental change in different islands;
4. contribute to clarify the potential occurrence of an "insect decline" in the Azores (see Borges et al. (2020)) and identify the spatial and temporal invasion patterns of exotic arthropod species (see Borges et al. (2022c));
5. contribute with temporal data to re-assess the IUCN Red-list status of Azorean endemic arthropods (Cardoso et al. 2011b);
6. perform studies about the relationship between diversity (taxonomic, functional and phylogenetic) and ecosystem functions.

### Additional information

**Figure 1.**
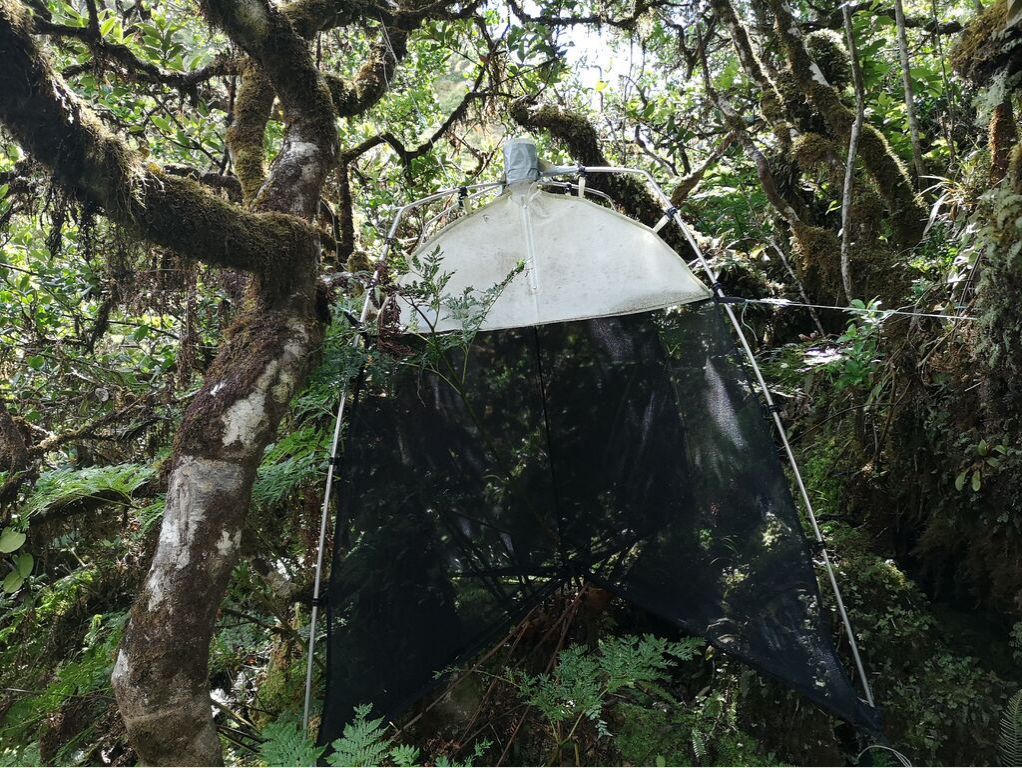
SLAM trap (Sea, Land and Air Malaise trap) located in a site on Terceira Island (Credit: Paulo A. V. Borges).

The year 2012 marks the beginning of the SLAM traps survey of arthropods on Terceira Island, within the Project NETBIOME ISLANDBIODIV. This first survey was then followed by several others within the Azores Archipelago with the purpose of sampling and describing all arthropods inside native forest fragments using passive SLAM traps (Sea, Land and Air Malaise trap, Fig. 1). During the last years, the data from these SLAM traps have been used to respond to several ecological and conservation questions (for example, see Matthews et al. (2018), Borges et al. (2020), de Vries et al. (2021), Tsafack et al. (In Press)).

## Project description

### Title

SLAM - Long Term Ecological Study of the Impacts of Climate Change in the natural forest of Azores.

### Personnel

The project was conceived and is being led by Paulo A.V. Borges

**Fieldwork**:

- Flores Island: Marlene Noia and Telma Figueiredo (Natural Park of Flores)
- Faial Island: Pedro Casimiro and João Bettencourt (Botanical Garden of Faial) and Cátia Freitas (Natural Park of Faial)
- Pico Island: Paulo Freitas and Sónia Manso (Natural Park of Pico)
- Graciosa Island: Carlos Picanço with the collaboration of Pedro Raposo (Natural Park of Graciosa)
- Terceira Island: Paulo A. V. Borges, Alejandra Ros-Prieto, Fernando Pereira, Lucas Lamelas-López, Rui Carvalho, Rui Nunes and Sébastien Lhoumeau.
- São Miguel Island: Miguel Ferreira (Natural Park of São Miguel) and Rúben Coelho (SPEA)
- Santa Maria Island: Nelson Moura (Natural Park of Santa Maria).

**Parataxonomists**:

For the period 2012-2019: Adal Humberto Díaz Raya, Adrian Fernandez Marinez, Alba Arteaga, Alejandra Ros-Prieto, Castore De Salvador, David Rodilla Rivas, Daniel Ehrhart, Elisa Tarantino, Gea Ghisolfi, Helena Marugán Páramo, Joel Martin Ay, Jonne Bonnet, Jose Vicente Pérez Santa Rita, Juan Ignacio Pitarch Peréz, Juan Manuel Taboada Alvarez, Laura Cáceres Sabater, Laura Gallardo, Magí Ramon Martorell, Maria Simitakou, Marija Tomašić, Marta Calera Sierra, Merili Martverk, Óscar García Contreras, Oscar Gomez Novillo, Percy de Laminne de Bex, Reinier Vries, Riccardo Negroni, Ruben Murillo Garcia, Rui Carvalho, Rui Nunes, Sébastien Lhoumeau, Sergio Fernandez, Sophie Wallon and William Razey.

For the period 2019-2021: Abrão Leite, Adrian Fernandez Marinez, Emanuela Cosma, Jonne Bonnet, Joel Martin Aye, Loïc Navarro, Magí Ramon Martorell, Marco Canino, Natalia Fierro Frerot, Sébastien Lhoumeau and Valentin Moley.

**Taxonomists**: Paulo A. V. Borges and Luís Carlos Crespo.

**Curation**: Voucher specimen management was mainly undertaken by Alejandra Ros-Prieto, Abrão Leite, Ricardo Costa, Sébastien Lhoumeau and Paulo A. V. Borges.

### Study area description

**Figure 2.**
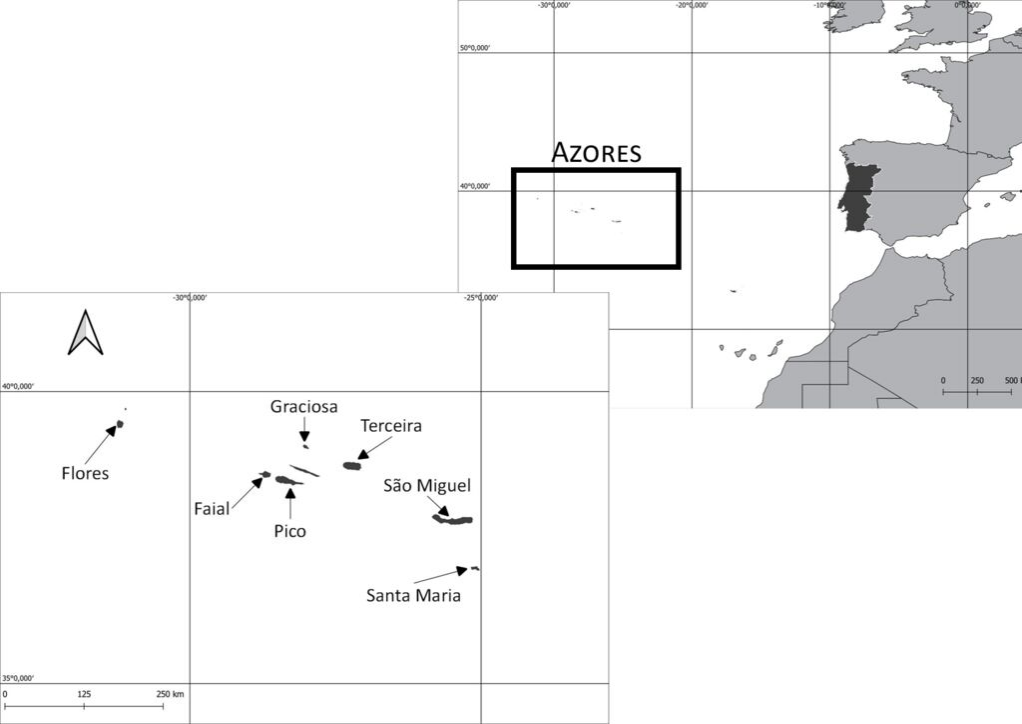
Location of the Azores Archipelago and the Islands of Flores, Faial, Graciosa, Pico, Terceira, São Miguel and Santa Maria.

The Azores Archipelago comprises nine volcanic Islands and is located in the Atlantic Ocean between latitudes 37° and 40° N (Fig. 2), situated in the mid-Atlantic Ocean spreading over 500 km in a W/NW–E/SE direction. All Islands are oceanic of recent volcanic origin and the prevalent climate is temperate, with no dry seasons and mild summers. Santa Maria and Graciosa are the driest Islands and the prevalent climate in these Islands is temperate with dry and warm summers.

**Figure 3.**
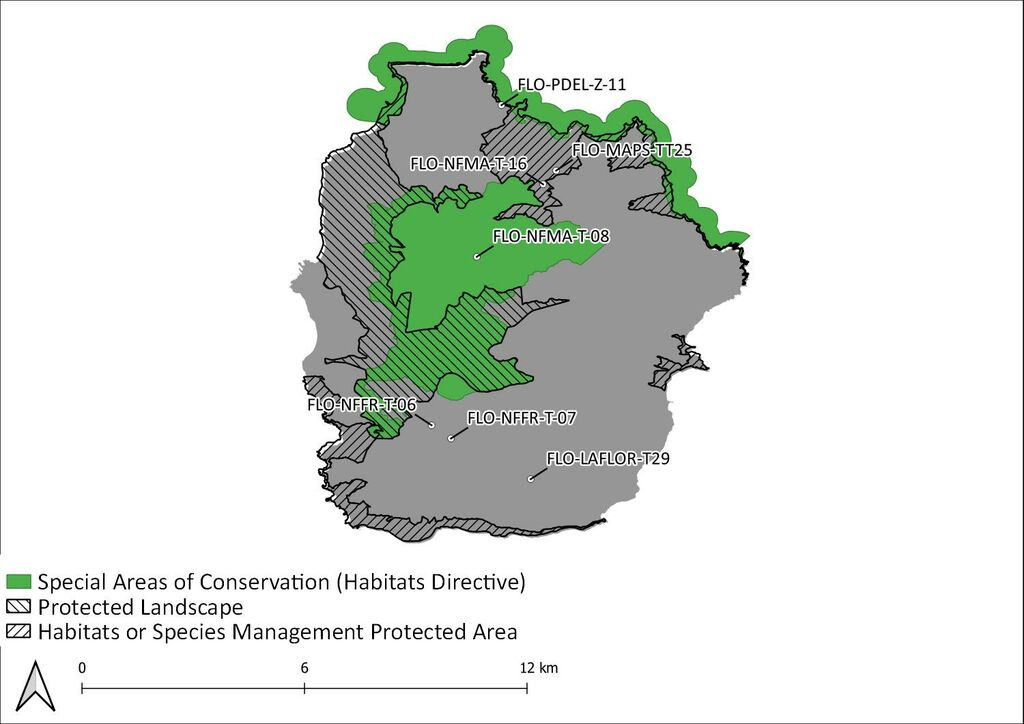
Flores Island: Protected areas and sampling areas are indicated. See complete site names in Table 1.

**Figure 4.**
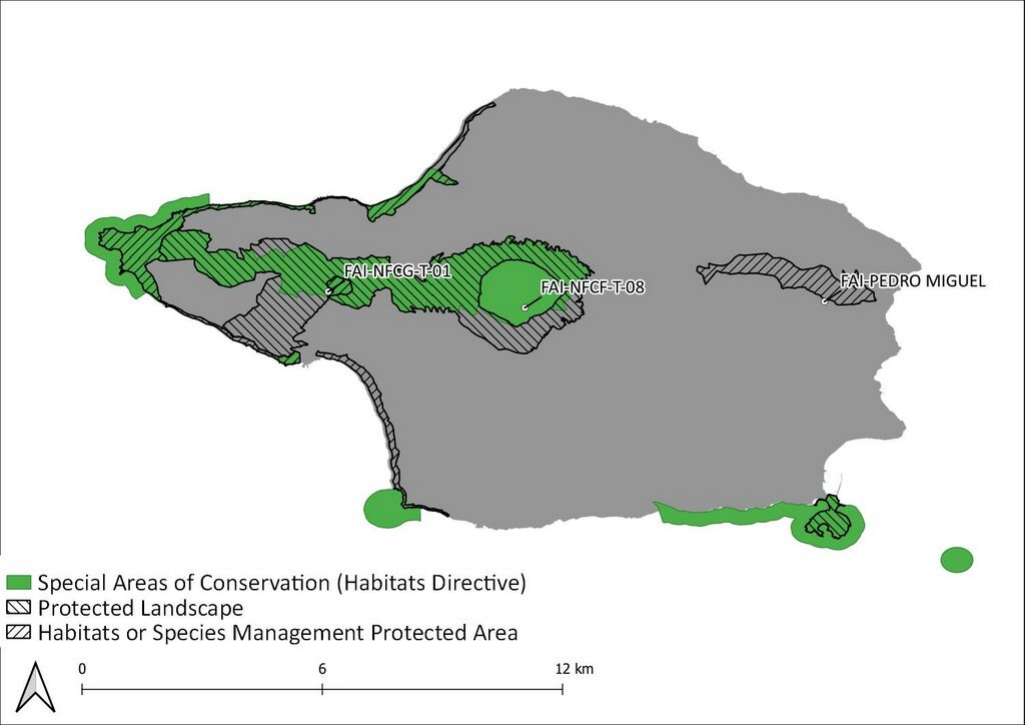
Faial Island: Protected areas and sampling areas are indicated. See complete site names in Table 1.

**Figure 5.**
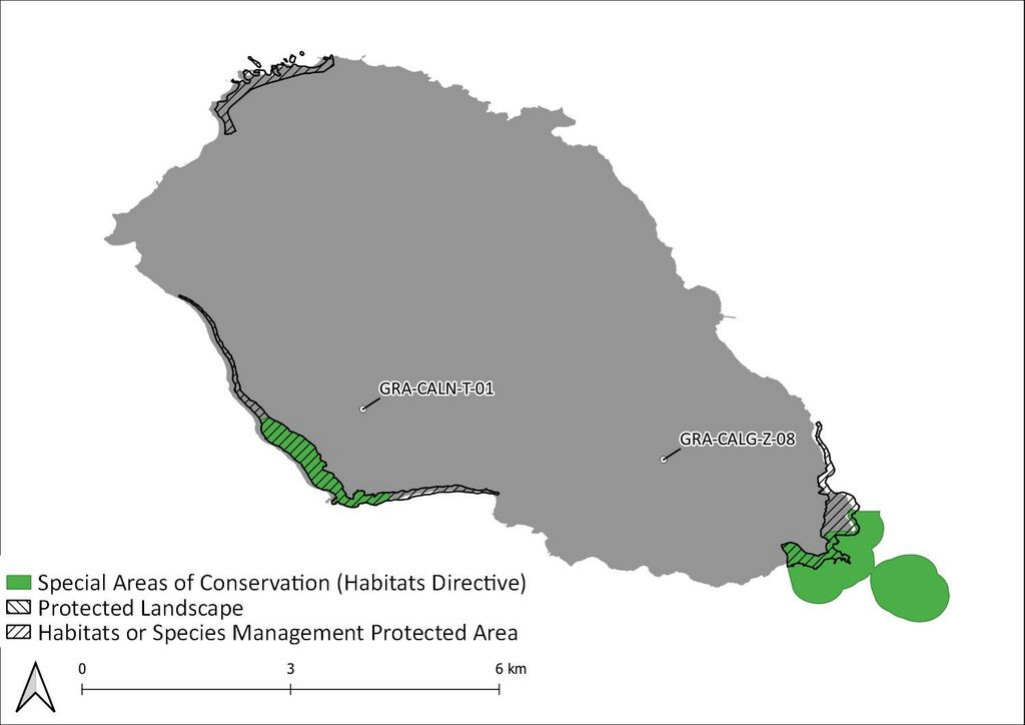
Graciosa Island: Protected areas and sampling areas are indicated. See complete site names in Table 1.

**Figure 6.**
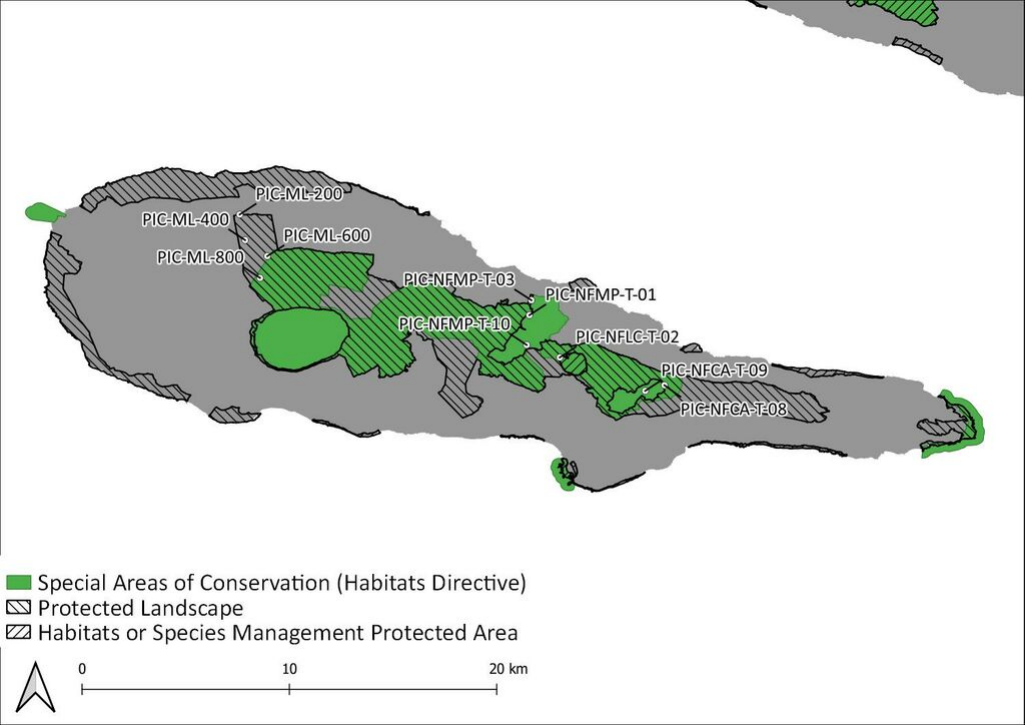
Pico Island: Protected areas and sampling areas are indicated. See complete site names in Table 1.

**Figure 7.**
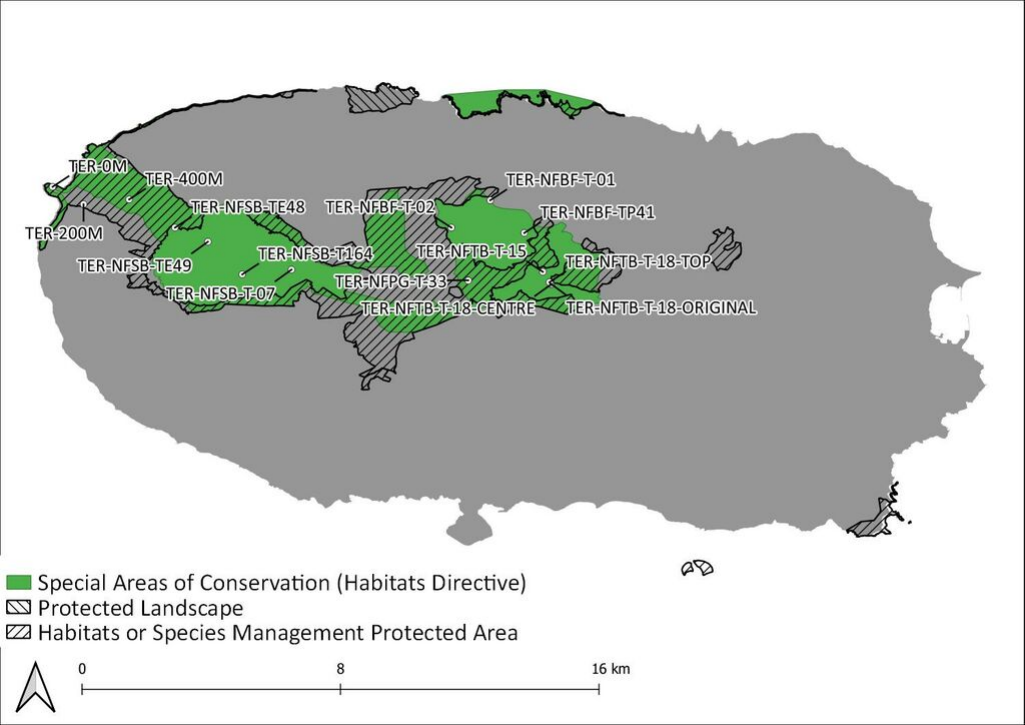
Terceira Island: Protected areas and sampling areas are indicated. See complete site names in Table 1.

**Figure 8.**
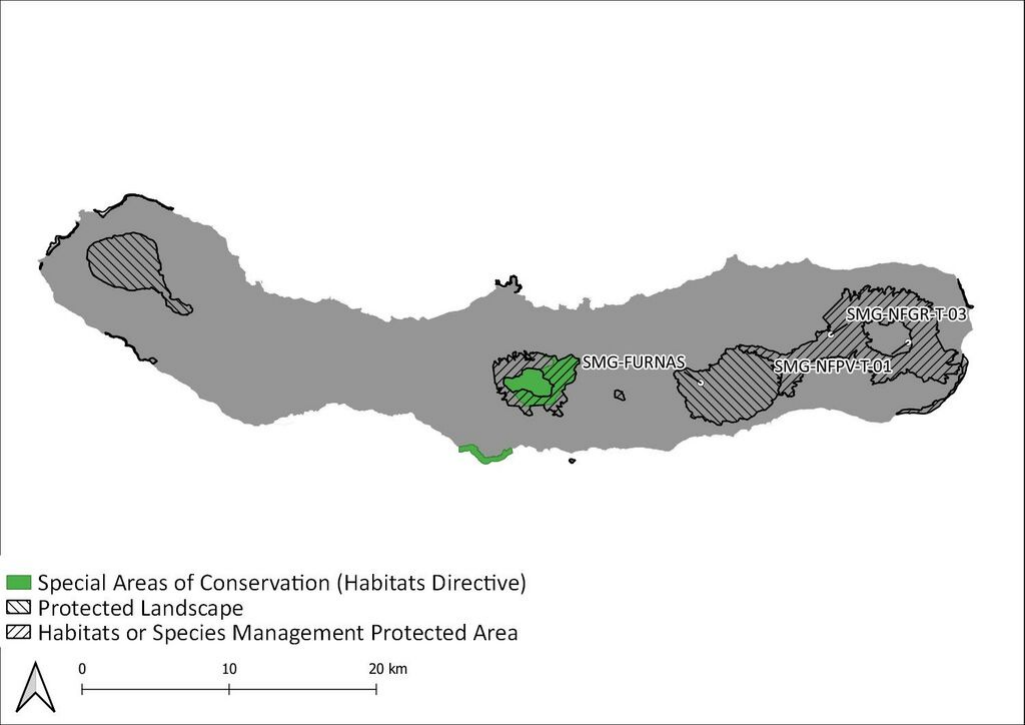
São Miguel Island: Protected areas and sampling areas are indicated. See complete site names in Table 1.

**Figure 9.**
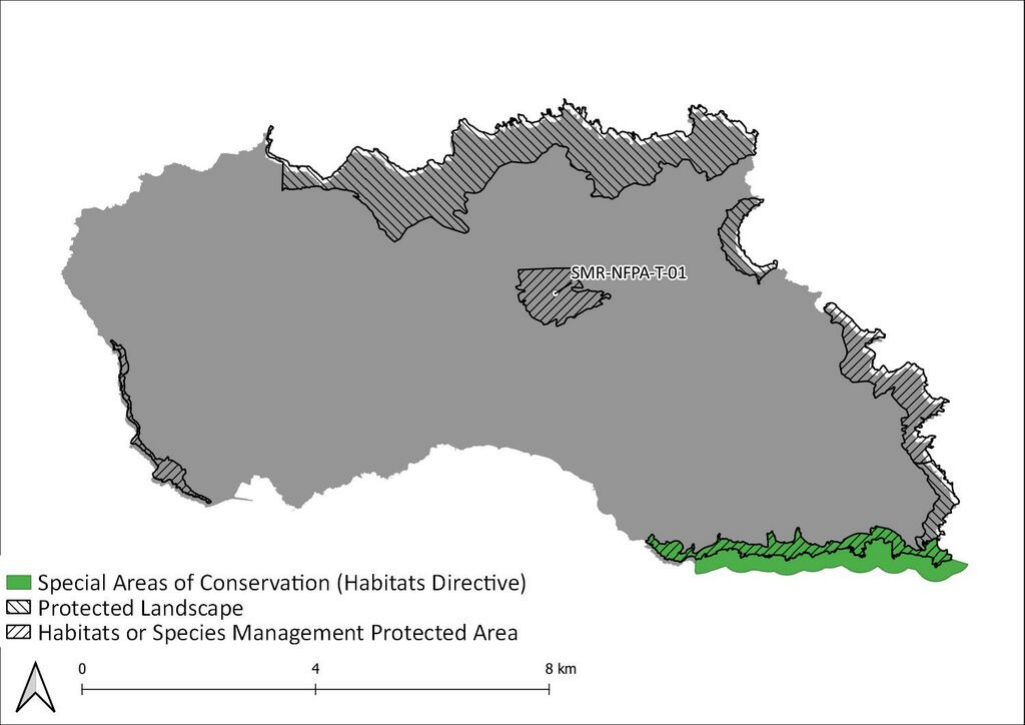
Santa Maria Island: Protected areas and sampling areas are indicated. See complete site names in Table 1.

During this project, seven Islands (Flores, Fig. 3; Faial, Fig. 4; Graciosa, Fig. 5; Pico, Fig. 6; Terceira, Fig. 7; São Miguel, Fig. 8 and Santa Maria, Fig. 9) were surveyed within the SLAM Project. The sampling areas (Table 1) are mostly dominated by endemic vegetation, but some sites are of mixed forest with the presence of invasive species like *Pittosporumundulatum* and *Hedychiumgardnerianum*.

**Figure 10.**
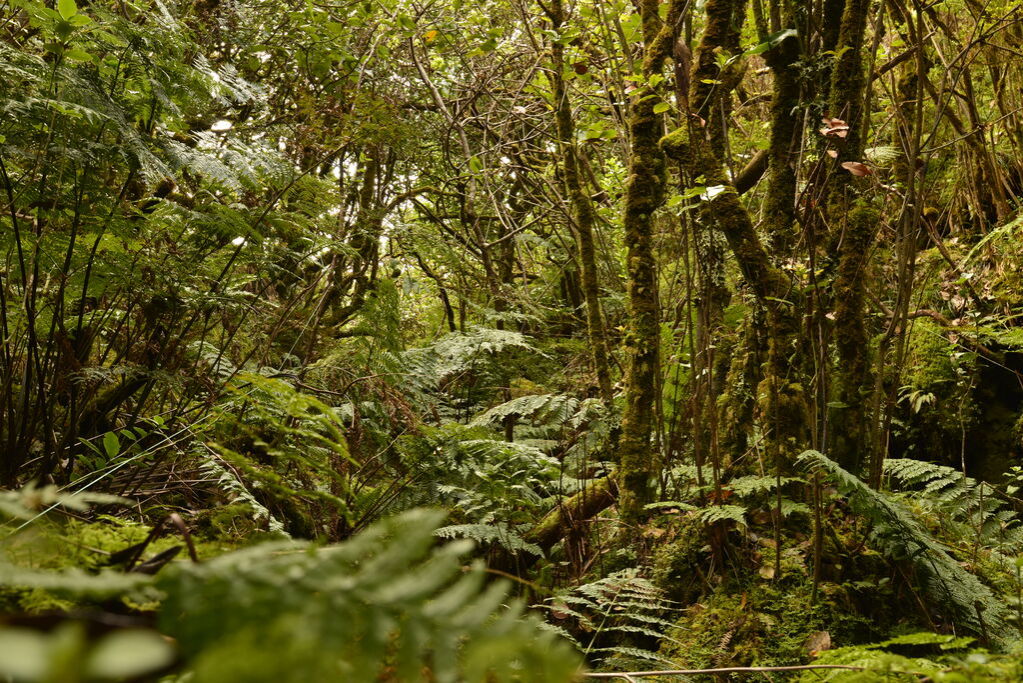
*Laurus* Submontane Forests in Terceira (TER-NFTB-T-18 - Terra Brava B) (Credit: Paulo A. V. Borges).

**Figure 11.**
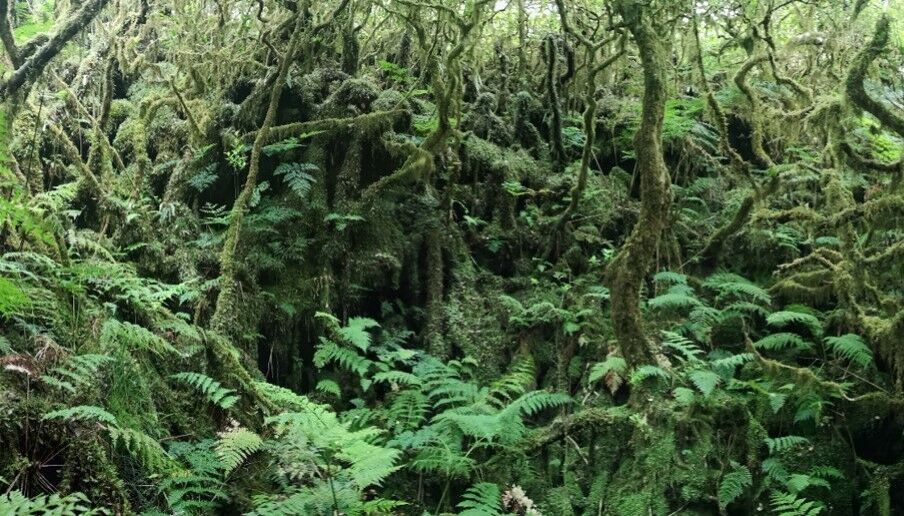
Dense cover of ferns and bryophytes in the native forest of Azores. *Juniperus*-*Ilex* Montane Forest in Mistério da Parinha at Pico Island (2020) (Credit: Paulo A. V. Borges).

Based on the recent forest classification proposal of Elias et al. (2016), most of the studied sites are located in the *Juniperus*-*Ilex* forests and *Juniperus* woodlands (between 600 m and 1000 m a.s.l.) (see Table 1), with some few remants of the *Laurus* Submontane Forests in Terceira (Fig. 10) and Pico Islands. All these forests are hyper-humid, densely covered by ferns and mosses at all strata (Fig. 11).

### Design description

We sampled on the Azorean Islands of Flores, Faial, Pico, Graciosa, Terceira, São Miguel and Santa Maria, four times per year between 2012 and 2021 (around the 15th March (winter sample), 15th June (spring sample), 15th September (summer sample) and 15th December (autumn sample).

However, on some Islands (e.g. Santa Maria and Graciosa) and sites (e.g. TER-NFTB-T-18 in Terceira in the period June 2014 - December 2015) (see Borges et al. (2022d)), samples were obtained every month for some years. The specimens collected were taken to the laboratory for identification and preservation and the resulting vouchers were deposited at the Dalberto Teixeira Pombo Insect Collection of the University of the Azores.

### Funding

- FCT-NETBIOME – ISLANDBIODIV grant 0003/2011 (between 2012 and 2015) with a funding of around 60 k euros.
- EU ERASMUS+ Training Grants to Ruben Murillo Garcia, Laura Gallardo (2014); Adal Humberto Díaz Raya, David Rodilla, Laura Cáceres Sabater, Óscar García Contrera, William Razey (2015); Alejandra Ros Prieto, Daniel Ehrhart, Helena Marugán Páramo, Maria Simitakou (2016); Juan Manuel Taboada Alvarez, Merili Martverk (2017); Elisa Tarantino, Marta Calera Sierra, Oscar Gomez-Novillo, Reinier Vries (2018); Adrian Fernandez Marinez, Castore De Salvador, Gea Ghisolfi, Joel Martin Aye, Riccardo Negroni (2019); Jonne Bonnet (2020), Magí Ramon Martorell, Sébastien Lhoumeau (2021), Emanuela Cosma, Loïc Navarro, Marco Canino, Valentin Moley (2022) with a total funding so far of around 90 k euros.
- EU EURODYSSÉE - Marija Tomašić (2014), Percy de Laminne de Bex, Juan Ignacio Pitarch Peréz (2015); Jose Vicente Pérez Santa Rita (2017); Alba Arteaga (2018), with a total funding so far of around 30 k euros.
- ESTAGIAR L Azores Government - Sophie Wallon (2014), with a funding of 12 k euros.
- ESTAGIAR T Azores Government - Alejandra Ros Prieto (2017), with a funding of 12 k euros.
- Portuguese National Funds, through FCT – Fundação para a Ciência e a Tecnologia, within the project UID/BIA/00329/2013-2020, with a funding of 9 k euros.
- Direcção Regional do Ambiente - PRIBES (LIFE17 IPE/PT/000010) (2019), with a funding of 6 k euros.
- Direcção Regional do Ambiente – LIFE-BETTLES (LIFE18 NAT_PT_000864) (2020), with a funding of 138 k euros until 2024.
- AZORESBIOPORTAL – PORBIOTA (ACORES-01-0145-FEDER-000072) (2019), with a funding of 9 k euros.
- Science and Technology Foundation (FCT) - MACRISK-Trait-based prediction of extinction risk and invasiveness for Northern Macaronesian arthropods (FCT-PTDC/BIA-CBI/0625/2021) with 9 k euros.
- Portal da Biodiversidade dos Açores (2022-2023) - PO Azores Project - M1.1.A/INFRAEST CIENT/001/2022 (2022).
- FCT-UIDB/00329/2020-2024 (Thematic Line 1 – integrated ecological assessment of environmental change on biodiversity) (2019-2022) with 3 k euros.

## Sampling methods

### Study extent

Overall, we sampled a total of 42 plots (seven in Flores, three in Faial, two in Graciosa, ten in Pico, 16 in Terceira, three in São Miguel and one in Santa Maria), using passive SLAM traps (Table 1). The plots are located in some of the best preserved wet forest patches of the seven Islands, having only limited human disturbance (Borges et al. 2017).

### Sampling description

We used passive flight interception SLAM traps (Sea, Land and Air Malaise trap; 110 x 110 x 110 cm) (MegaView Science Co. Ltd., Taichung City, Taiwan) (Fig. 1) to sample native forest plots in several Azorean Islands, with one trap placed at each plot.

The trapped arthropods crawl up the mesh and then fall inside the sampling recipient. Each recipient is filled with propylene glycol (pure 1,2-propanediol) to kill the captured arthropods and conserve the sample between collections, enabling also the preservation of DNA for future genetic analyses. Although this protocol was developed to sample flying arthropods, by working as an extension of the tree, non-flying species, such as spiders, can also crawl into the trap, widening the range of groups that can be sampled by this technique.

### Quality control

In the laboratory, specimen sorting and arthropod identification followed standard procedures, using somatic and genitalic features for species identification. A reference collection was made for all collected specimens (whether or not identified at species level) by assigning them a morphospecies code number and depositing them at the Dalberto Teixeira Pombo Insect Collection (DTP), University of Azores (Terceira Island).

## Geographic coverage

### Description

Flores, Faial, Pico, Graciosa, Terceira, São Miguel and Santa Maria Islands in the Azores, Macaronesia, Portugal (Fig. 2).

### Coordinates

36.844 and 39.690 Latitude; -31.333 and -24.785 Longitude.

## Taxonomic coverage

### Description

The following classes and orders are covered:

Arachnida: Araneae, Opiliones, Pseudoscorpiones

Chilopoda: Geophilomorpha, Lithobiomorpha, Scolopendromorpha, Scutigeromorpha

Diplopoda: Chordeumatida, Julida, Polydesmida

Insecta: Archaeognatha, Blattodea, Coleoptera, Dermaptera, Ephemeroptera, Hemiptera, Hymenoptera (Formicidae), Neuroptera, Orthoptera, Phasmida, Psocodea, Strepsiptera, Thysanoptera, Trichoptera

Symphyla: Symphyla

## Traits coverage

Functional traits of Araneae including detailed morphometric measurements for most of the studied species can be accessed in the publication by Macías-Hernández et al. (2020).

Trophic preference for all other arthropods are assessed using the publication by Rigal et al. (2018).

## Temporal coverage

### Notes

**Figure 12.**
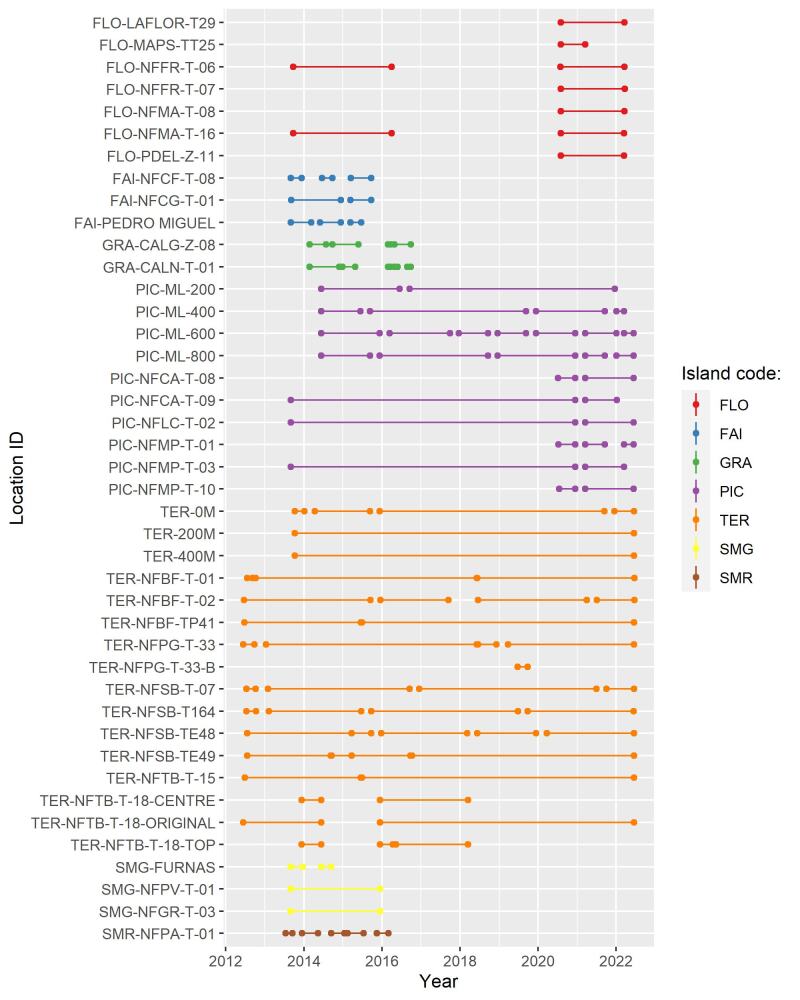
Temporal coverage of each plot. Codes of sites as in Table 1.

Despite our efforts, not all islands could be continuously monitored. The temporal graph hereafter (Fig. 12) shows the range of temporal coverage for all traps.

## Usage licence

### Usage licence

Creative Commons Public Domain Waiver (CC-Zero)

## Data resources

### Data package title

Long-term monitoring of Azorean forest arthropods

### Resource link

http://ipt.gbif.pt/ipt/resource?r=arthropods_slam_azores

### Alternative identifiers

https://www.gbif.org/dataset/079c8358-0b4f-479b-97dd-1f2f775256f9

### Number of data sets

2

### Data set 1.

#### Data set name

Event table

#### Data format

Darwin Core Archive format

#### Character set

UTF-8

#### Download URL

http://ipt.gbif.pt/ipt/resource?r=arthropods_slam_azores

#### Data format version

Version 1.3

#### Description

The dataset was published in the Global Biodiversity Information Facility platform, GBIF (Borges and Lhoumeau 2022). The following data table includes all the records for which a taxonomic identification of the species was possible. The dataset submitted to GBIF is structured as a sample event dataset that has been published as a Darwin Core Archive (DwCA), which is a standardised format for sharing biodiversity data as a set of one or more data tables. The core data file contains 893 records (eventID). This GBIF IPT (Integrated Publishing Toolkit, Version 2.5.6) archives the data and, thus, serves as the data repository. The data and resource metadata are available for download in the Portuguese GBIF Portal IPT (Borges and Lhoumeau 2022).

**Data set 1.** 

Column label	Column description
id	Unique identification code for sampling event data.
eventID	Identifier of the events, unique for the dataset.
samplingProtocol	The sampling protocol used to capture the species.
sampleSizeValue	The numeric amount of time spent in each sampling (in days).
sampleSizeUnit	The unit of the sample size value.
eventDate	Date or date range the record was collected.
eventRemarks	The verbatim original representation of the date and time information for an Event. In this case, we use the season and year.
habitat	The habitat from which the sample was obtained.
locationID	Identifier of the location.
islandGroup	Name of archipelago, always Azores in the dataset.
island	Name of the island.
country	Country of the sampling site, always Portugal in the dataset.
countryCode	ISO code of the country of the sampling site, always PT in the dataset.
stateProvince	Name of the region of the sampling site.
municipality	Municipality of the sampling site.
locality	Name of the locality.
minimumElevationInMetres	The lower limit of the range of elevation (altitude, above sea level), in metres.
locationRemarks	Details on the locality site.
decimalLatitude	Approximate decimal latitude of the trap.
decimalLongitude	Approximate decimal longitude of the trap.
geodeticDatum	The ellipsoid, geodetic datum or spatial reference system (SRS) upon which the geographic coordinates given in decimalLatitude and decimalLongitude are based, always WGS84 in the dataset.
coordinateUncertaintyInMetres	Uncertainty of the coordinates of the centre of the sampling plot.
coordinatePrecision	Precision of the coordinates.
georeferenceSources	A list (concatenated and separated) of maps, gazetteers or other resources used to georeference the Location, described specifically enough to allow anyone in the future to use the same resources.

### Data set 2.

#### Data set name

Occurrence table

#### Data format

Darwin Core Archive format

#### Character set

UTF-8

#### Download URL

http://ipt.gbif.pt/ipt/resource?r=arthropods_slam_azores

#### Data format version

Version 1.3

#### Description

The dataset was published in the Global Biodiversity Information Facility platform, GBIF (Borges and Lhoumeau 2022). The following data table includes all the records for which a taxonomic identification of the species was possible. The dataset submitted to GBIF is structured as an occurrence table that has been published as a Darwin Core Archive (DwCA), which is a standardised format for sharing biodiversity data as a set of one or more data tables. The core data file contains 14824 records (occurrenceID). This GBIF IPT (Integrated Publishing Toolkit, Version 2.5.6) archives the data and, thus, serves as the data repository. The data and resource metadata are available for download in the Portuguese GBIF Portal IPT (Borges and Lhoumeau 2022).

**Data set 2.** 

Column label	Column description
id	Unique identification code for species abundance data. Equivalent here to eventID.
type	The nature or genre of the resource, as defined by the Dublin Core standard. In our case "PhysicalObject".
licence	Reference to the licence under which the record is published.
institutionID	The identity of the institution publishing the data.
collectionID	The identity of the collection publishing the data.
institutionCode	The code of the institution publishing the data.
collectionCode	The code of the collection where the specimens are conserved.
datasetName	Name of the dataset.
basisOfRecord	The nature of the data record.
recordedBy	A list (concatenated and separated) of names of people, groups or organisations who performed the sampling in the field.
occurrenceID	Identifier of the record, coded as a global unique identifier.
organismQuantity	A number or enumeration value for the quantity of organisms.
organismQuantityType	The type of quantification system used for the quantity of organisms.
sex	The sex and quantity of the individuals captured.
lifeStage	The life stage of the organisms captured.
establishmentMeans	The process of establishment of the species in the location, using a controlled vocabulary: 'native', 'introduced', 'endemic', 'indeterminate'.
eventID	Identifier of the events, unique for the dataset.
identifiedBy	A list (concatenated and separated) of names of people, groups or organisations who assigned the taxon to the subject.
dateIdentified	The date on which the subject was determined as representing the taxon.
scientificName	Complete scientific name including author and year.
kingdom	Kingdom name.
phylum	Phylum name.
class	Class name.
order	Order name.
family	Family name.
genus	Genus name.
specificEpithet	Specific epithet.
infraspecificEpithet	Infraspecific epithet.
taxonRank	Lowest taxonomic rank of the record.
scientificNameAuthorship	Name of the author of the lowest taxon rank included in the record.
identificationRemarks	Information about morphospecies identification (code in Dalberto Teixeira Pombo Collection).

## Additional information

We collected a total of 176007 specimens from which 168565 (95.7%) were identified at species or subspecies level. These identified specimens belong to 25 orders, 106 families, 260 genera and 316 species or subspecies. In this pool of 316 named species and subspecies, a total of 132 species are considered introduced, 88 native non-endemic, 55 endemic and 41 have indeterminate colonisation status.

Based on a comparison with the previous Azorean arthropod checklist (Borges et al. 2010), we recorded a total of 72 unique new records at Island level (Table 2) . None of these records is new for the Azores Archipelago as they were already sampled in other monitoring programmes for other Islands.

Details on the new records for the Islands:

**Two new species records for Flores Island (Table 3)**:

- One beetle (Coleoptera, Leiodidae), *Catopscoracinus* Kellner, 1846 (native non-endemic), that is a saprophagous species commonly found in several habitats in Azores (native and exotic forests, entrance of caves).

- One thrips (Thysanoptera, Thripidae), *Ceratothripsericae* (Haliday, 1836) (native non-endemic), that is usually associated with the endemic shrub *Ericaazorica*.

**Eight new species records for Faial Island (Table 3)**:

- Two spiders (Araneae, Theridiidae), *Neottiurabimaculata* (Linnaeus, 1767) and *Theridionmelanurum* Hahn, 1831, both introduced and very common in human-made habitats.

- Four beetles (Coleoptera), *Clitostethusarcuatus* (Rossi, 1794) (introduced), *Dryopsalgiricus* (Lucas, 1846) (native non-endemic), *Notothectadryochares* (Israelson, 1985) (endemic) and *Scymnussuturalis* Thunberg, 1795 (introduced). *C.arcuatus* and *S.suturalis* are ladybeetles (Coccinellidae) widespread in Azores (Soares et al. 2021). *Notothectadryochares* (Staphylinidae) is the most abundant endemic rove-beetle in Azores (Borges et al. 2022a), commonly found associated with the canopy and trunks of endemic trees.

- Two bugs (Hemiptera), *Brachystelesparvicornis* (A. Costa, 1847) (Anthocoridae) (native non-endemic) and *Eupteryxazorica* Ribaut, 1941 (Cicadellidae) (endemic). *B.parvicornis* is a common predator mostly found in human-made habitats and *E.azorica* is commonly associated with native and endemic ferns in native forest.

**Twenty new species records for Graciosa Island (Table 3)**:

- Five Araneae: *Chalcoscirtusinfimus* (Simon, 1868) (introduced), *Lathysdentichelis* (Simon, 1883) (native non-endemic), *Macaroerisdiligens* (Blackwall, 1867) (native non-endemic), *Pachygnathadegeeri* Sundevall, 1830 (introduced) and *Pelecopsisparallela* (Wider, 1834) (introduced). *C.infimus* and *M.diligens* are both jumping spiders (Salticidae) very common in exotic forests, gardens and orchards. *L.dentichelis* (Dictynidae) is one of the most common spiders in the canopies of endemic trees in Azores. *P.degeeri* (Tetragnathidae) is mosltly associated with humid areas like margins of lakes, but can also be found in pastures. *P.parallela* (Linyphiidae) is widely distributed in Azorean pastures.

- Eleven beetles (Coleoptera): *Aspidapionradiolus* (Marsham, 1802) (Apionidae) (introduced), *Cryptophaguscellaris* (Scopoli, 1763) (Cryptophagidae) (introduced), *Dromiusmeridionalis* Dejean, 1825 (Carabidae) (introduced), *Kalcapionsemivittatumsemivittatum* (Gyllenhal, 1833) (Apionidae) (indeterminate), *Longitarsuskutscherai* (Rye, 1872) (Chrysomelidae) (introduced), *Mecinuspascuorum* (Gyllenhal, 1813) (Curculionidae) (introduced), *Naupactusleucoloma* Boheman, 1840 (Curculionidae) (introduced), *Proteinusatomarius* Erichson, 1840 (Staphylinidae) (indeterminate), *Stenomastaxmadeirae* Assing, 2003 (Staphylinidae) (indeterminate), *Tachyporusnitidulus* (Fabricius, 1781) (Staphylinidae) (indeterminate) and *Trichiusarobustula* Casey, 1893 (Staphylinidae) (indeterminate). It is particularly relevant to mention the fact that most of these beetle species are exotic historically introduced species.

- Five bugs (Hemiptera): *Euphylluraolivina* (Costa, 1839) (Liviidae) (introduced), *Loriculacoleoptrata* (Fallén, 1807) (Microphysidae) (native non-endemic), *Piezodoruslituratus* (Fabricius, 1794) (Pentatomidae) (native non-endemic), *Plinthisusminutissimus* Fieber 1864, (Rhyparochromidae) (native non-endemic) and *Siphantaacuta* (Walker, 1851) (Flatidae) (introduced). Particularly relevant the presence of *S.acuta*, that is spreading fast in Azores (see also Borges et al. (2013)).

- Three Psocodea: *Bertkauialucifuga* (Rambur, 1842) (Epipsocidae) (native non-endemic), *Trichadenotecnumcastum* Betz, 1983 (Psocidae) (introduced), *Valenzuelaburmeisteri* (Brauer, 1876) (Caeciliusidae) (native non-endemic). All these species are common in many forest habitats of all islands in Azores.

**Twenty-three new species records for Pico Island (Table 3)**:

- One centipede (Chilopoda, Geophilomorpha, Linotaeniidae), *Strigamiacrassipes* (C.L. Koch, 1835) (native non-endemic).

- Four millipedes (Diplopoda), all introduced in Azores: *Brachyiuluspusillus* (Leach, 1814) (Julidae), *Cylindroiuluslatestriatus* (Curtis, 1845) (Julidae), *Nopoiuluskochii* (Gervais, 1847) (Blaniulidae) and *Proteroiulusfuscus* (Am Stein, 1857) (Blaniulidae).

- Eight beetles (Coleoptera): *Catopscoracinus* Kellner, 1846 (Leiodidae) (native non-endemic), *Charagmusgressorius* (Fabricius, 1792) (Curculionidae) (native non-endemic), *Cryptophaguscellaris* (Scopoli, 1763) (Cryptophagidae) (introduced), *Mecinuspascuorum* (Gyllenhal, 1813) (Curculionidae) (introduced), *Medonapicalis* (Kraatz, 1857) (Staphylinidae) (indeterminated), *Rhizophagusferrugineus* (Paykull, 1800) (Monotomidae) (introduced), *Sitonadiscoideus* Gyllenhal, 1834 (Curculionidae) (introduced) and *Stilbustestaceus* (Panzer, 1797) (Phalacridae) (native non-endemic).

- Six bugs (Hemiptera): *Anthocorisnemoralis* (Fabricius, 1794) (Anthocoridae) (native non-endemic), *Geotomuspunctulatus* (A. Costa, 1847) (Cydnidae) (native non-endemic), *Lasiosomusenervis* (Herrich-Schäffer, 1835) (Rhyparochromidae) (native non-endemic), *Loriculacoleoptrata* (Fallén, 1807) (Microphysidae) (native non-endemic), *Plinthisusminutissimus* Fieber, 1864 (Rhyparochromidae) (native non-endemic) and *Siphantaacuta* (Walker, 1851) (Flatidae) (introduced). Similarly to Graciosa, *S.acuta* is also new for Pico, species that is spreading fast in Azores (Borges et al. 2013).

- Two Orthoptera: *Eumodicogryllusbordigalensis* (Latreille, 1804) (Gryllidae) (introduced) and *Phaneropteranana* Fieber, 1853 (Tettigoniidae) (native non-endemic).

- Two native non-endemic Psocodea: *Bertkauialucifuga* (Rambur, 1842) (Epipsocidae) and *Valenzuelaburmeisteri* (Brauer, 1876) (Caeciliusidae).

**Eight new species records for Terceira Island (Table 3)**:

- Three beetles (Coleoptera): *Aleocharaclavicornis* L. Redtenbacher, 1849 (Chrysomelidae) (indeterminate), *Metophthalmusoccidentalis* Israelson, 1984 (Latridiidae) (endemic), *Philonthusquisquiliariusquisquiliarius* (Gyllenhal, 1810) (Staphylinidae) (indeterminate).

- Five native non-endemic bugs (Hemiptera): *Loriculacoleoptrata* (Fallén, 1807) (Microphysidae), *Loriculaelegantula* (Bärensprung, 1858) (Microphysidae), *Microplaxplagiatus* (Fieber, 1837) (Microphysidae), *Miridiusquadrivirgatus* (A. Costa, 1853) (Oxycarenidae) and *Piezodoruslituratus* (Fabricius, 1794) (Pentatomidae).

**Three new species records for São Miguel Island (Table 3)**:

- One endemic spider (Araneae, Linyphiidae), *Canariphantesacoreensis* (Wunderlich, 1992).

- Two beetles (Coleoptera): *Cyphaseminulum* (Erichson, 1839) (Staphylinidae) (indeterminate) and *Longitarsuskutscherai* (Chrysomelidae) (Rye, 1872) (introduced).

**Four new species records for Santa Maria Island (Table 3)**:

- One introduced spider (Araneae, Linyphiidae), *Nerieneclathrata* (Sundevall, 1830).

- Two beetles (Coleoptera): *Catopscoracinus* Kellner, 1846 (Leiodidae) (native non-endemic) and *Cryptophaguscellaris* (Scopoli, 1763) (Cryptophagidae) (introduced).

- One endemic bug (Hemiptera, Cicadellidae), *Eupteryxazorica* Ribaut, 1941, usually associated with native and endemic ferns.

### Conservation remarks

We recorded 23 introduced species which are new to the Islands of the Azores Archpelago. This number of new records is higher than for the endemic (n = 4), native (n = 21) and indeterminate (n = 8) species. These new records increase the diversity of the species at island scale. They must be considered with particular attention as they might rapidly increase their distribution. However, we need to be careful with these new records because they could represent an effect of the past low sampling effort and not recent introductions. Indeed, not all Islands were sampled with the same intensity through time. In order to provide better time series analysis, we must continue sampling arthropods over all Islands with increasing regularity.

**Figure 13.**
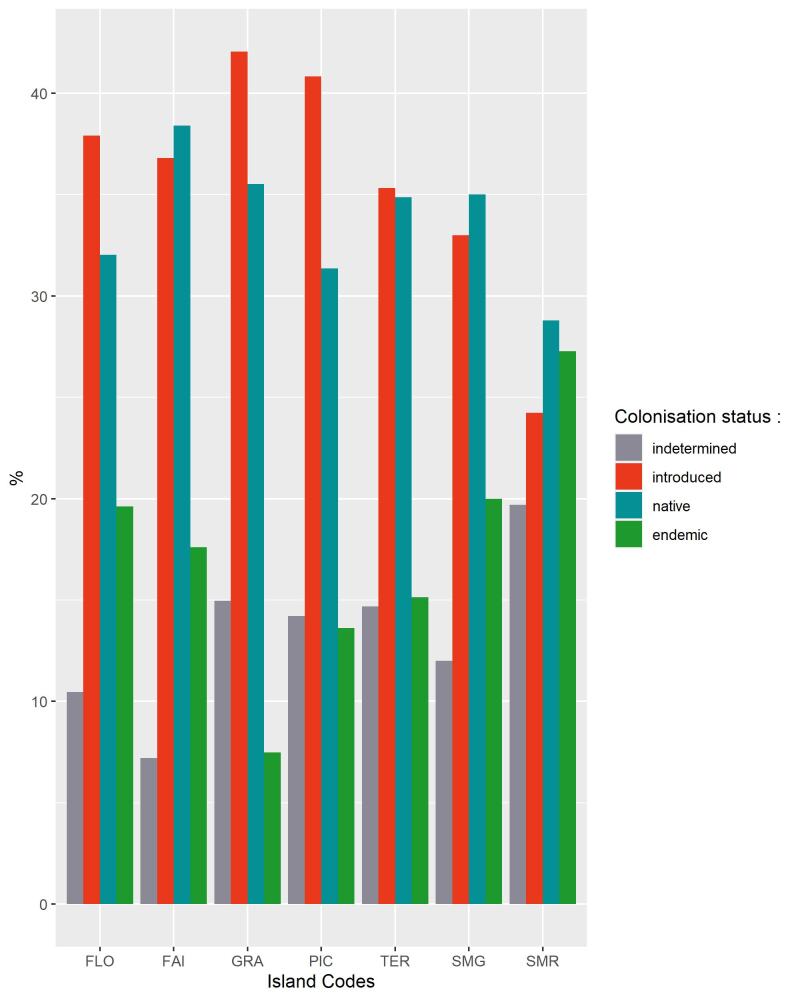
Bar plot of percentage of number of species according to their colonisation status for each Island (FLO-Flores; FAI – Faial; GRA – Graciosa; PIC – Pico; TER – Terceira; SMG – São Miguel; SMR – Santa Maria).

Introduced species are the greatest part of the new records, but also the most diverse group of species over all the Archipelago (Fig. 13). In all Islands we monitored with this programme, introduced species represent almost 25% of the species richness of a given Island. Graciosa (42%), Pico (41%) and Flores (38%) are the three Islands with the highest percentage of introduced species sampled (Fig. 13). It is a strong and critical signal for Graciosa as this is the Island where the temporal coverage of sampling is the lowest (Fig. 12), but with the highest proportion of introduced species. This result must be the consequence of the limited amount of native forest on this Island. Indeed, there is no fragment of native forest remaining on this Island, only a small secondary patch dominated by the endemic tree species *Ericaazorica*, as an early succession shrub (see Species of Habitat management areas in Fig. 5). Furthermore, Graciosa Island is the second smallest island of the Archipelago (after Corvo Island) and the lowest in altitude. All these parameters can explain that the quality of the fragment where we sampled is poor in comparison to other Islands (like Terceira Island, where the greatest fragments of native forest occur) (Triantis et al. 2010). Indeed, other long term monitoring studies performed in non-native environments (Borges et al. 2013, Borges et al. 2022a, Borges et al. 2022b) showed that it is more likely to find exotic arthropods species in such disturbed places. Therefore, in Graciosa, monitoring programmes should be encouraged to obtain time series of arthropod communities so that trends can be detected and forecast. In any case, particular attention must be given to exotic species as they are part of the global biodiversity crisis experienced in the Archipelago (Borges et al. 2019, Borges et al. 2020, Singh 2002).

**Figure 14.**
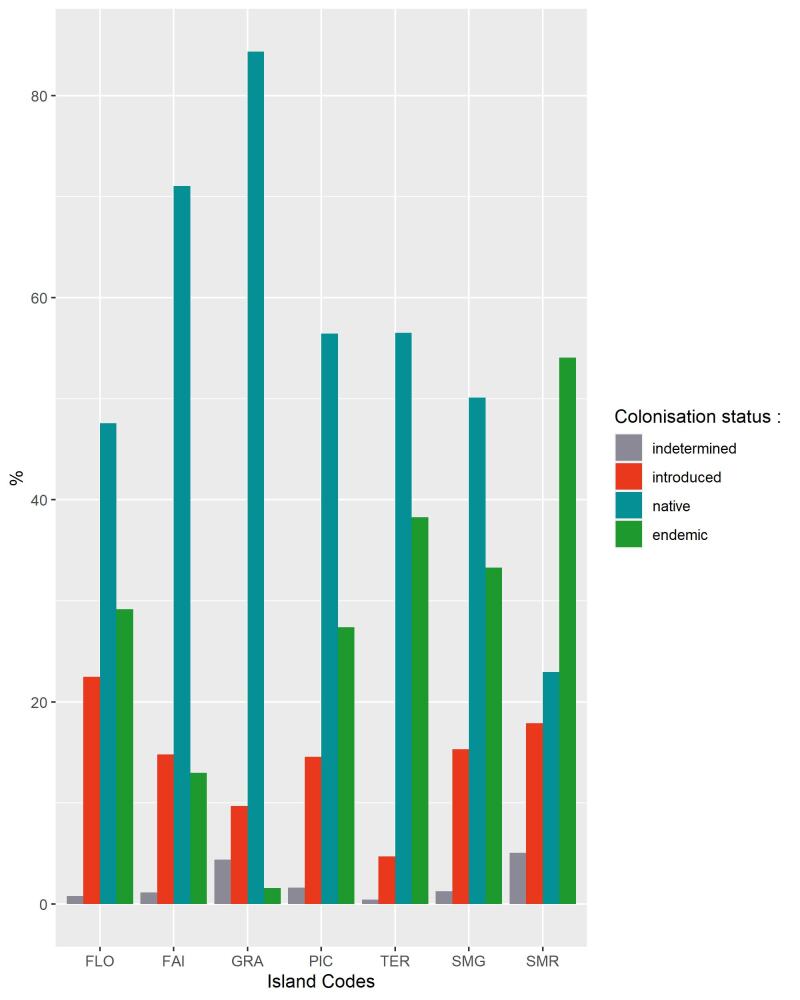
Bar plot of percentage of abundance of species according to their colonisation status for each Island (FLO – Flores; FAI – Faial; GRA – Graciosa; PIC – Pico; TER – Terceira; SMG – São Miguel; SMR – Santa Maria).

A positive output of this study is the non-dominance of introduced species in native forest patches, a result that coincides with a recent study conducted on a native forest fragment Terra-Brava on Terceira (see Borges et al. (2017)). Fig. 14 shows that almost all of the seven Islands we monitored have less than 20% of their total species abundance composed of introduced species (except for Flores Island where 22% of the total abundance of arthropods sampled are introduced). However, we detected that the highest proportion of the abundance in Graciosa Island is attributed to native non-endemic species. Further analysis is needed to explore links between such dominance of native non-endemic species and the quality of the habitat. Variations of the proportion of exotic species amongst the Islands are likely to be the consequence of difference of habitat quality, age and size of the Island and human activities and interaction between these factors (Borges et al. 2006).

All of the seven Islands monitored showed that introduced arthropod species are the most diverse group, but not the dominant one, which suggest that introduced species are mostly vagrants with large turnover rates across space and time. Such turnover on oceanic islands is common to other taxa like plants (Gilbert and Lechowicz 2005, Kueffer et al. 2010). A future analysis of beta diversity drivers through time can shed light on the dynamics of invasions on these Islands (Carvalho et al. 2012, Legendre and De Cáceres 2013). Finally, our results show that the forest fragments where we performed our samplings are likely to be resistant to an increasing pressure of the constant introduction of exotic species (but see Borges et al. (2020)). However, monitoring needs to be continued to detect the crossing of a potentially dramatic threshold (Xie et al. 2019) leading to profound and irreversible changes in the composition and functioning of native Azorean ecosystems.

## Figures and Tables

**Table 1.** List of the 42 sampled sites in Flores (n = 7), Faial (n = 3), Graciosa (n = 2), Pico (n = 10), Terceira (n = 16), São Miguel (n = 3) and Santa Maria (n = 1) Islands. Information about Location ID, Locality, decimal coordinates and elevation in metres are provided. In the habitat, we classify the type of forest, based in Elias et al. (2016): (1) *Erica*-*Morella* Coastal Woodlands, (2) *Picconia*-*Morella* Lowland Forests, (3) *Laurus* Submontane Forests, (4) *Juniperus*-*Ilex* Montane Forests, (5) *Juniperus* Montane Woodlands.

Island	Habitat	Location ID	Locality	Latitude	Longitude	Elevation
Flores	Mixed forest	FLO-LAFLOR-T29	Lajes - Estação florestal	39.39053	-31.19257	278
Flores	Exotic forest	FLO-MAPS-TT25	Criptomérias ao lado do T15	39.48697	-31.18462	618
Flores	Native forest (5)	FLO-NFFR-T-06	Juniperal	39.40736	-31.22355	499
Flores	Native forest (4)	FLO-NFFR-T-07	Encosta Caldeira Funda	39.40324	-31.21750	376
Flores	Native forest (5)	FLO-NFMA-T-08	Morro Alto Este	39.46003	-31.20941	766
Flores	Native forest (5)	FLO-NFMA-T-16	Ribeira do Cascalho	39.48269	-31.18868	642
Flores	Mixed forest	FLO-PDEL-Z-11	Km18 - Mata de acácias	39.50744	-31.20170	98
Faial	Native forest (5)	FAI-NFCF-T-08	Fora Trilho da Caldeira	38.58119	-28.71291	725
Faial	Native forest (1)	FAI-NFCG-T-01	Erical	38.58580	-28.76921	416
Faial	Native forest (5)	FAI-PEDRO-MIGUEL	Pedro Miguel	38.58298	-28.62712	225
Graciosa	Erica forest (1)	GRA-CALN-T-01	Calderinha de Pêro Botelho	39.03841	-28.03039	348
Graciosa	Mixed forest	GRA-CALG-Z-08	Caldeira da Graciosa-Furna da Maria Encantada	39.03003	-27.98049	227
Pico	Mixed forest	PIC-ML-200	Plot 200m	38.53480	-28.43410	199
Pico	Mixed forest	PIC-ML-400	Plot 400m	38.52070	-28.43110	428
Pico	Mixed forest	PIC-ML-600	Plot 600m	38.51190	-28.41890	627
Pico	Mixed forest	PIC-ML-800	Plot 800m	38.49990	-28.42290	797
Pico	Native forest (5)	PIC-NFCA-T-08	Picos do Caveiro	38.44082	-28.20001	888
Pico	Native forest (5)	PIC-NFCA-T-09	Caveiro Base	38.43770	-28.21060	937
Pico	Native forest (4)	PIC-NFLC-T-02	Euphorbias	38.45610	-28.25770	804
Pico	Native forest (3)	PIC-NFMP-T-01	Chão Verde superior	38.47937	-28.27444	612
Pico	Native forest (3)	PIC-NFMP-T-03	Chão Verde inferior	38.48760	-28.27330	475
Pico	Native forest (4)	PIC-NFMP-T-10	Mistério da Prainha Zona Trilho ddos Burros à esquerda.	38.46298	-28.27593	778
Terceira	Native forest (1)	TER-0M	Farol da Serreta	38.76660	-27.37480	46
Terceira	Mixed forest (2)	TER-200M	Serreta 200m	38.76040	-27.36380	237
Terceira	Mixed forest (2)	TER-400M	Miradouro do Pico Carneiro	38.76210	-27.34760	397
Terceira	Native forest (3)	TER-NFBF-T-01	Labaçal - Morro Assombrado	38.76180	-27.21930	678
Terceira	Native forest (5)	TER-NFBF-T-02	Chambre A	38.75210	-27.23310	590
Terceira	Native forest (5)	TER-NFBF-TP41	Pico Alto Nascente	38.75020	-27.20720	673
Terceira	Native forest (4)	TER-NFPG-T-33	Pico X B	38.73340	-27.22710	642
Terceira	Native forest (4)	TER-NFPG-T-33-B	Pico X B_B	38.73332	-27.22711	642
Terceira	Native forest (4)	TER-NFSB-T-07	Lomba	38.73720	-27.28990	683
Terceira	Native forest (5)	TER-NFSB-T164	Caldeira - Silvia	38.73550	-27.30740	900
Terceira	Native forest (5)	TER-NFSB-TE48	Lagoinha B	38.75210	-27.33130	687
Terceira	Native forest (5)	TER-NFSB-TE49	Lagoa Pinheiro B	38.74710	-27.31960	918
Terceira	Native forest (3)	TER-NFTB-T-15	Terra Brava A	38.73640	-27.20060	637
Terceira	Native forest (3)	TER-NFTB-T-18-CENTRE	Terra Brava B - Centre	38.73195	-27.19770	682
Terceira	Native forest (3)	TER-NFTB-T-18-ORIGINAL	Terra Brava B - Original	38.73235	-27.19798	686
Terceira	Native forest (3)	TER-NFTB-T-18-TOP	Terra Brava B - Top	38.73272	-27.19827	684
São Miguel	Exotic forest	SMG-FURNAS	Parque Furnas	37.76560	-25.34448	511
São Miguel	Native forest (4)	SMG-NFGR-T-03	Encosta	37.80250	-25.24438	921
São Miguel	Native forest (4)	SMG-NFPV-T-01	Miradouro da Tronqueira	37.79674	-25.18491	653
Santa Maria	Native forest (2)	SMR-NFPA-T-01	Pico Alto STM A	36.98024	-25.09079	563

**Table 2.** List of arthropod species collected in seven Islands of Azores, between 2012 and 2022 using SLAM traps. The list includes individuals identified at species-level. Scientific name, colonisation status (CS: I – introduced; N - native non-endemic; E – endemic; NA - indeterminate) and abundance per Island (FAI - Faial; FLO - Flores; GRA – Graciosa; PIC – Pico; SMG – São Miguel; TER - Terceira; SMR - Santa Maria). Bold scientific names constitute the ten most abundant species for the Azores and numbers with stars are new records for a given island. Data for Araneae from Pico and Terceira Islands are not mentioned because they were already made available in Costa and Borges (2021)

Class	Order	Scientific name	CS	FLO	FAI	PIC	GRA	TER	SMG	SMR
Arachnida	Araneae	*Acorigoneacoreensis* (Wunderlich, 1992)	E	65	24	---	0	---	0	0
Arachnida	Araneae	*Agalenatearedii* (Scopoli, 1763)	I	1	0	---	0	---	0	0
Arachnida	Araneae	*Agynetadepigmentata* Wunderlich, 2008	E	1	0	---	0	---	0	0
Arachnida	Araneae	*Agynetafuscipalpa* (C. L. Koch, 1836)	I	0	1	---	2	---	0	0
Arachnida	Araneae	*Canariphantesacoreensis* (Wunderlich, 1992)	E	3	1	---	0	---	2*	0
Arachnida	Araneae	*Canariphantesjunipericola* Crespo & Bosmans, 2014	E	3	0	---	0	---	0	0
Arachnida	Araneae	*Chalcoscirtusinfimus* (Simon, 1868)	I	0	0	---	1*	---	0	0
Arachnida	Araneae	*Cheiracanthiumerraticum* (Walckenaer, 1802)	I	0	6	---	0	---	0	2
Arachnida	Araneae	*Cheiracanthiumfloresense* Wunderlich, 2008	E	75	0	---	0	---	0	0
Arachnida	Araneae	*Cheiracanthiummildei* L. Koch, 1864	I	15	0	---	0	---	0	0
Arachnida	Araneae	*Clubionaterrestris* Westring, 1851	I	3	0	---	0	---	0	0
Arachnida	Araneae	*Cryptachaeablattea* (Urquhart, 1886)	I	2	7	---	76	---	36	4
Arachnida	Araneae	*Drassodeslapidosus* (Walckenaer, 1802)	I	0	1	---	0	---	0	0
Arachnida	Araneae	*Dysderacrocata* C. L. Koch, 1838	I	71	0	---	2	---	0	0
Arachnida	Araneae	*Emblynaacoreensis* Wunderlich, 1992	E	3	0	---	9	---	0	0
Arachnida	Araneae	*Erigoneatra* Blackwall, 1833	I	0	0	---	1	---	1	0
Arachnida	Araneae	*Erigonedentipalpis* (Wider, 1834)	I	0	1	---	0	---	2	0
Arachnida	Araneae	*Eroaphana* (Walckenaer, 1802)	I	1	0	---	0	---	0	0
Arachnida	Araneae	*Erofurcata* (Villers, 1789)	I	10	3	---	1	---	0	0
Arachnida	Araneae	*Gibbaraneaoccidentalis* Wunderlich, 1989	E	199	34	---	6	---	16	35
Arachnida	Araneae	*Lasaeolaoceanica* Simon, 1883	E	1	2	---	0	---	0	3
Arachnida	Araneae	*Lathysdentichelis* (Simon, 1883)	N	149	14	---	4*	---	17	8
Arachnida	Araneae	*Leucognathaacoreensis* Wunderlich, 1992	E	38	42	---	0	---	82	13
Arachnida	Araneae	*Macaroeriscata* (Blackwall, 1867)	N	18	12	---	0	---	3	0
Arachnida	Araneae	*Macaroerisdiligens* (Blackwall, 1867)	N	8	1	---	4*	---	0	0
Arachnida	Araneae	*Mermessusfradeorum* (Berland, 1932)	I	0	1	---	0	---	2	0
Arachnida	Araneae	*Microlinyphiajohnsoni* (Blackwall, 1859)	N	0	2	---	0	---	0	0
Arachnida	Araneae	*Miniciafloresensis* Wunderlich, 1992	E	0	0	---	0	---	0	0
Arachnida	Araneae	*Neonacoreensis* Wunderlich, 2008	E	3	1	---	0	---	0	0
Arachnida	Araneae	*Neottiurabimaculata* (Linnaeus, 1767)	I	0	1*	---	0	---	2	0
Arachnida	Araneae	*Nerieneclathrata* (Sundevall, 1830)	I	3	0	---	0	---	3	1*
Arachnida	Araneae	*Nigmapuella* (Simon, 1870)	I	3	0	---	0	---	0	0
Arachnida	Araneae	*Oecobiussimilis* Kulczynski, 1909	N	0	0	---	6	---	0	0
Arachnida	Araneae	*Oedothoraxfuscus* (Blackwall, 1834)	I	0	0	---	1	---	1	0
Arachnida	Araneae	*Pachygnathadegeeri* Sundevall, 1830	I	1	0	---	4*	---	1	0
Arachnida	Araneae	*Palliduphantesschmitzi* (Kulczynski, 1899)	N	3	1	---	0	---	0	0
Arachnida	Araneae	*Pardosaacorensis* Simon, 1883	E	3	0	---	0	---	1	0
Arachnida	Araneae	*Pelecopsisparallela* (Wider, 1834)	I	0	0	---	22*	---	0	0
Arachnida	Araneae	*Pholcommagibbum* (Westrung, 1851)	I	1	0	---	0	---	0	0
Arachnida	Araneae	*Pisauraacoreensis* Wunderlich, 1992	E	20	20	---	0	---	1	0
Arachnida	Araneae	*Porrhoclubionadecora* (Blackwall, 1859)	N	1	35	---	36	---	1	0
Arachnida	Araneae	*Porrhoclubionagenevensis* (L. Koch, 1866)	I	37	1	---	0	---	0	0
Arachnida	Araneae	*Prinerigonevagans* (Audouin, 1826)	I	0	0	---	0	---	1	0
Arachnida	Araneae	*Pseudeuophrysvafra* (Blackwall, 1867)	I	1	0	---	0	---	0	0
Arachnida	Araneae	*Rugathodesacoreensis* Wunderlich, 1992	E	125	129	---	0	---	220	35
Arachnida	Araneae	*Salticusmutabilis* Lucas, 1846	I	2	0	---	1	---	0	0
Arachnida	Araneae	*Savigniorrhipisacoreensis* Wunderlich, 1992	E	618	37	---	0	---	43	17
Arachnida	Araneae	*Steatodagrossa* (C. L. Koch, 1838)	I	1	3	---	3	---	0	1
Arachnida	Araneae	*Steatodanobilis* (Thorell, 1875)	N	4	3	---	0	---	4	2
Arachnida	Araneae	*Tegenariapagana* C. L. Koch, 1840	I	0	1	---	0	---	0	0
Arachnida	Araneae	*Tenuiphantesmiguelensis* (Wunderlich, 1992)	N	31	37	---	1	---	9	8
Arachnida	Araneae	*Tenuiphantestenuis* (Blackwall, 1852)	I	98	574	---	72	---	48	3
Arachnida	Araneae	*Tetragnathaextensa* (Linnaeus, 1758)	I	0	0	---	0	---	1	0
Arachnida	Araneae	*Textrixcaudata* L. Koch, 1872	I	75	0	---	0	---	0	0
Arachnida	Araneae	*Theridionmelanurum* Hahn, 1831	I	0	1*	---	0	---	0	0
Arachnida	Araneae	*Theridionmusivivum* Schmidt, 1956	N	1	7	---	0	---	0	0
Arachnida	Araneae	*Walckenaeriagrandis* (Wunderlich, 1992)	E	0	0	---	0	---	0	0
Arachnida	Araneae	*Xysticuscor* Canestrini, 1873	N	3	9	---	9	---	0	0
Arachnida	Araneae	*Xysticusnubilus* Simon, 1875	I	0	1	---	0	---	0	0
Arachnida	Opiliones	*Homalenotuscoriaceus* (Simon, 1879)	N	109	3	51	0	14	5	0
Arachnida	Opiliones	***Leiobunumblackwalli* Meade, 1861**	N	402	708	10939	209	9034	673	0
Arachnida	Pseudoscorpiones	*Chthoniusischnocheles* (Hermann, 1804)	I	0	0	68	8	117	0	0
Arachnida	Pseudoscorpiones	*Ephippiochthoniustetrachelatus* (Preyssler, 1790)	I	0	0	13	0	108	0	0
Arachnida	Pseudoscorpiones	*Neobisiummaroccanum* Beier, 1930	I	118	149	2644	2	25	0	0
Chilopoda	Geophilomorpha	*Geophilustruncorum* Bergsøe & Meinert, 1866	N	0	0	1	0	0	0	0
Chilopoda	Geophilomorpha	*Strigamiacrassipes* (C.L. Koch, 1835)	N	0	0	110*	0	2	1	0
Chilopoda	Lithobiomorpha	*Lithobiuspilicornispilicornis* Newport, 1844	N	254	5	160	0	461	0	0
Chilopoda	Scolopendromorpha	*Cryptiopshortiensis* (Donovan, 1810)	N	0	0	0	0	2	1	0
Chilopoda	Scutigeromorpha	*Scutigeracoleoptrata* (Linnaeus, 1758)	I	20	0	46	23	641	0	0
Diplopoda	Chordeumatida	*Haplobainosomalusitanum* Verhoeff, 1900	I	0	0	84	0	57	1	0
Diplopoda	Julida	*Blaniulusguttulatus* (Fabricius, 1798)	I	5	0	12	0	0	0	0
Diplopoda	Julida	*Brachyiuluspusillus* (Leach, 1814)	I	5	1	4*	0	0	0	0
Diplopoda	Julida	*Cylindroiuluslatestriatus* (Curtis, 1845)	I	1	0	13*	0	0	0	0
Diplopoda	Julida	*Cylindroiuluspropinquus* (Porat, 1870)	I	1	1	200	0	6	1	0
Diplopoda	Julida	*Nopoiuluskochii* (Gervais, 1847)	I	1	0	7*	0	2	0	0
Diplopoda	Julida	***Ommatoiulusmoreleti* (Lucas, 1860)**	I	680	261	1927	85	818	0	3
Diplopoda	Julida	*Proteroiulusfuscus* (Am Stein, 1857)	I	1	1	26*	0	1	0	0
Diplopoda	Polydesmida	*Brachydesmussuperus* Latzel, 1884	I	0	0	7	0	23	0	0
Diplopoda	Polydesmida	*Oxidusgracilis* (C.L. Koch, 1847)	I	20	18	15	0	0	0	0
Diplopoda	Polydesmida	*Polydesmuscoriaceus* Porat, 1870	I	5	0	45	1	4	0	0
Insecta	Archaeognatha	*Diltasaxicola* (Womersley, 1930)	N	22	0	1182	92	598	0	0
Insecta	Archaeognatha	***Trigoniophthalmusborgesi* Mendes, Gaju, Bach & Molero, 2000**	E	0	4	116	0	5011	0	0
Insecta	Blattodea	*Zethasimonyi* (Krauss, 1892)	N	143	46	242	0	1892	5	32
Insecta	Coleoptera	*Acupalpusdubius* Schilsky, 1888	N	0	0	0	0	2	0	0
Insecta	Coleoptera	*Acupalpusflavicollis* (Sturm, 1825)	N	0	0	0	0	1	0	0
Insecta	Coleoptera	*Aeolusmelliculusmoreleti* Tarnier, 1860	I	0	0	0	0	1	0	0
Insecta	Coleoptera	*Agonummuellerimuelleri* (Herbst, 1784)	I	0	0	0	0	1	0	0
Insecta	Coleoptera	*Aleocharabipustulata* (Linnaeus, 1760)	NA	6	1	0	3	77	2	8
Insecta	Coleoptera	*Aleocharaclavicornis* L. Redtenbacher, 1849	NA	0	0	0	0	1*	0	0
Insecta	Coleoptera	*Aleocharafunebris* Wollaston, 1864	NA	1	0	0	0	0	0	0
Insecta	Coleoptera	*Alestrusdolosus* (Crotch, 1867)	E	1	0	0	0	2	1	0
Insecta	Coleoptera	*Aloconotasulcifrons* (Stephens, 1832)	NA	1	0	4	5	7	0	3
Insecta	Coleoptera	*Amaraaenea* (De Geer, 1774)	I	0	0	0	0	3	0	0
Insecta	Coleoptera	*Amischaanalis* (Gravenhorst, 1802)	NA	1	0	0	0	2	0	0
Insecta	Coleoptera	*Amischaforcipata* Mulsant & Rey, 1873	NA	0	0	0	0	1	0	0
Insecta	Coleoptera	*Anaspisproteus* Wollaston, 1854	N	80	60	295	60	463	22	3
Insecta	Coleoptera	*Anisodactylusbinotatus* (Fabricius, 1787)	I	0	0	2	0	5	0	0
Insecta	Coleoptera	*Anobiumpunctatum* (De Geer, 1774)	I	1	0	3	4	76	0	0
Insecta	Coleoptera	*Anommatusduodecimstriatus* (Müller, 1821)	I	0	0	27	0	0	2	0
Insecta	Coleoptera	*Anotylusnitidulus* (Gravenhorst, 1802)	NA	0	0	1	0	0	0	0
Insecta	Coleoptera	*Aspidapionradiolus* (Marsham, 1802)	I	0	10	2	1*	29	1	0
Insecta	Coleoptera	*Astenuslyonessius* (Joy, 1908)	NA	1	16	0	6	6	0	0
Insecta	Coleoptera	*Athetaaeneicollis* (Sharp, 1869)	NA	7	0	13	11	53	5	8
Insecta	Coleoptera	*Athetaatramentaria* (Gyllenhal, 1810)	NA	0	3	2	0	3	1	1
Insecta	Coleoptera	*Athetafungi* (Gravenhorst, 1806)	NA	1	4	46	32	15	3	26
Insecta	Coleoptera	*Athouspomboi* Platia & Borges, 2002	E	0	0	0	0	0	0	6
Insecta	Coleoptera	*Atlantocisgillerforsi* Israelson, 1985	E	0	0	88	0	17	0	0
Insecta	Coleoptera	*Brassicogethesaeneus* (Fabricius, 1775)	I	0	1	9	0	4	0	0
Insecta	Coleoptera	*Calacallessubcarinatus* (Israelson, 1984)	E	289	8	868	1	703	1	4
Insecta	Coleoptera	*Carpelimuscorticinus* (Gravenhorst, 1806)	NA	1	0	6	1	2	2	5
Insecta	Coleoptera	*Carpelimuszealandicus* (Sharp, 1900)	I	1	0	0	0	0	0	0
Insecta	Coleoptera	*Carpophilusfumatus* Boheman, 1851	I	0	0	0	0	1	0	0
Insecta	Coleoptera	*Cartoderebifasciata* (Reitter, 1877)	I	0	8	0	0	10	0	0
Insecta	Coleoptera	*Cartoderenodifer* (Westwood, 1839)	I	0	2	41	9	10	13	0
Insecta	Coleoptera	*Cartoderesatelles* (Blackburn, 1888)	I	0	0	0	0	1	0	0
Insecta	Coleoptera	*Catopscoracinus* Kellner, 1846	N	153*	37	22*	10	167	86	6*
Insecta	Coleoptera	*Catopsvelhocabrali* Blas & Borges, 1999	E	0	0	0	0	0	0	18
Insecta	Coleoptera	*Cercyonhaemorrhoidalis* (Fabricius, 1775)	I	0	0	15	1	23	0	2
Insecta	Coleoptera	*Chaetocnemahortensis* (Fourcroy, 1785)	I	3	27	5	0	0	1	1
Insecta	Coleoptera	*Charagmusgressorius* (Fabricius, 1792)	N	2	0	5*	0	4	1	0
Insecta	Coleoptera	*Chrysolinabankii* (Fabricius, 1775)	N	0	0	2	0	5	1	0
Insecta	Coleoptera	*Chrysolinahyperici* (Forster, 1771)	I	0	0	0	1	0	0	0
Insecta	Coleoptera	*Clitostethusarcuatus* (Rossi, 1794)	I	0	1*	0	0	8	0	0
Insecta	Coleoptera	*Coccotrypescarpophagus* (Hornung, 1842)	I	0	0	2	1	23	0	0
Insecta	Coleoptera	*Coproporuspulchellus* (Erichson, 1839)	NA	0	0	1	0	2	0	0
Insecta	Coleoptera	*Cordaliaobscura* (Gravenhorst, 1802)	NA	0	0	1	0	6	0	0
Insecta	Coleoptera	*Creophilusmaxillosusmaxillosus* (Linnaeus, 1758)	NA	0	0	0	0	1	0	0
Insecta	Coleoptera	*Crotchiellabrachyptera* Israelson, 1985	E	0	0	17	0	2	0	0
Insecta	Coleoptera	*Cryptamorphadesjardinsii* (Guérin-Méneville, 1844)	I	2	0	3	0	3	1	0
Insecta	Coleoptera	*Cryptophaguscellaris* (Scopoli, 1763)	I	1	0	22*	2*	6	0	3*
Insecta	Coleoptera	*Cyphaseminulum* (Erichson, 1839)	NA	0	0	0	0	0	1*	0
Insecta	Coleoptera	*Dromiusmeridionalis* Dejean, 1825	I	0	2	6	8*	26	0	0
Insecta	Coleoptera	*Drouetiusborgesiborgesi* (Machado, 2009)	E	0	0	0	0	344	0	0
Insecta	Coleoptera	*Dryopsalgiricus* (Lucas, 1846)	N	4	2*	1	0	1	0	0
Insecta	Coleoptera	*Dryopsluridus* (Erichson, 1847)	N	0	1	4	0	1	0	0
Insecta	Coleoptera	*Epitrixcucumeris* (Harris, 1851)	I	0	0	1	6	2	1	5
Insecta	Coleoptera	*Epitrixhirtipennis* (Melsheimer, 1847)	I	1	0	0	1	1	0	0
Insecta	Coleoptera	*Epuraeabiguttata* (Thunberg, 1784)	I	3	0	1	0	0	1	2
Insecta	Coleoptera	*Euconnusazoricus* Franz, 1969	E	0	0	43	0	0	0	0
Insecta	Coleoptera	*Euplectusinfirmus* Raffray, 1910	NA	0	49	535	0	14	0	0
Insecta	Coleoptera	*Gabriusnigritulus* (Gravenhorst, 1802)	NA	0	0	4	2	2	0	1
Insecta	Coleoptera	*Gonipterusplatensis* (Marelli, 1926)	I	0	0	0	0	1	0	0
Insecta	Coleoptera	*Heteroderesazoricus* (Tarnier, 1860)	E	70	0	0	0	20	0	0
Insecta	Coleoptera	*Heteroderesvagus* Candèze, 1893	I	6	0	0	0	24	0	0
Insecta	Coleoptera	*Holoparamecuscaularum* (Aubé, 1843)	I	0	0	2	0	0	0	0
Insecta	Coleoptera	*Hydroporusguernei* Régimbart, 1891	E	0	0	0	0	2	0	0
Insecta	Coleoptera	*Ischnopterapionvirens* (Herbst, 1797)	I	0	0	1	0	0	0	0
Insecta	Coleoptera	*Kalcapionsemivittatumsemivittatum* (Gyllenhal, 1833)	NA	1	0	1	1*	4	0	0
Insecta	Coleoptera	*Laemostenuscomplanatus* (Dejean, 1828)	I	0	2	4	0	2	0	0
Insecta	Coleoptera	*Litargusbalteatus* LeConte, 1856	I	0	0	1	0	0	0	0
Insecta	Coleoptera	*Longitarsuskutscherai* (Rye, 1872)	I	186	26	113	41*	15	6*	0
Insecta	Coleoptera	*Mecinuspascuorum* (Gyllenhal, 1813)	I	0	0	3*	9*	3	0	0
Insecta	Coleoptera	*Medonapicalis* (Kraatz, 1857)	NA	0	0	1*	0	0	0	0
Insecta	Coleoptera	*Melanotusdichrous* (Erichson, 1841)	I	2	0	0	0	4	0	0
Insecta	Coleoptera	*Metophthalmusoccidentalis* Israelson, 1984	E	0	0	0	1	1*	0	0
Insecta	Coleoptera	*Microlestesnegritanegrita* (Wollaston, 1854)	N	0	0	0	0	1	0	0
Insecta	Coleoptera	*Naupactuscervinus* (Boheman, 1840)	I	0	1	26	7	41	0	0
Insecta	Coleoptera	*Naupactusleucoloma* Boheman, 1840	I	0	0	0	6*	0	0	0
Insecta	Coleoptera	*Notothectadryochares* (Israelson, 1985)	E	8	1*	89	0	545	38	23
Insecta	Coleoptera	*Noviuscardinalis* (Mulsant, 1850)	I	9	0	1	0	6	1	0
Insecta	Coleoptera	*Ocypusaethiops* (Waltl, 1835)	NA	0	0	0	0	17	0	0
Insecta	Coleoptera	*Ocypusolens* (Müller, 1764)	NA	0	0	15	0	0	0	0
Insecta	Coleoptera	*Ocysharpaloides* (Audinet-Serville, 1821)	N	6	1	59	1	0	0	0
Insecta	Coleoptera	*Oligotapumilio* Kiesenwetter, 1858	NA	1	0	3	1	4	0	0
Insecta	Coleoptera	*Orthochaetesinsignis* (Aubé, 1863)	N	1	0	0	0	0	0	0
Insecta	Coleoptera	*Otiorhynchuscribricollis* Gyllenhal, 1834	I	6	3	8	0	3	0	2
Insecta	Coleoptera	*Otiorhynchusrugosostriatus* (Goeze, 1777)	I	0	0	1	0	10	0	0
Insecta	Coleoptera	*Otiorhynchussulcatus* (Fabricius, 1775)	I	7	1	0	0	0	0	0
Insecta	Coleoptera	*Paranchusalbipes* (Fabricius, 1796)	I	0	0	5	0	51	0	0
Insecta	Coleoptera	*Paraphloeostibagayndahensis* (MacLeay, 1871)	I	0	1	0	0	1	1	0
Insecta	Coleoptera	*Philonthuslongicornis* Stephens, 1832	NA	1	0	0	0	0	0	0
Insecta	Coleoptera	*Philonthusquisquiliariusquisquiliarius* (Gyllenhal, 1810)	NA	0	0	0	0	1*	0	0
Insecta	Coleoptera	*Phloeonomuspunctipennis* Thomson, 1867	NA	0	0	6	0	1	0	1
Insecta	Coleoptera	*Phloeoporacorticaliscorticalis* (Gravenhorst, 1802)	NA	0	0	1	0	0	0	0
Insecta	Coleoptera	*Phloeosinusgillerforsi* Bright, 1987	E	0	0	2	0	11	0	0
Insecta	Coleoptera	*Phloeostibaazorica* (Fauvel, 1900)	E	0	0	7	0	0	0	0
Insecta	Coleoptera	*Pissodescastaneus* (DeGeer, 1775)	I	0	0	12	0	0	0	0
Insecta	Coleoptera	*Platystethusnitens* (Sahlberg, 1832)	NA	0	0	0	0	0	1	0
Insecta	Coleoptera	*Popilliajaponica* Newman, 1838	I	1	0	1	0	1	0	0
Insecta	Coleoptera	*Proteinusatomarius* Erichson, 1840	NA	1	0	35	3*	44	4	2
Insecta	Coleoptera	*Psapharochrusjaspideus* (Germar, 1824)	I	0	0	1	0	0	0	0
Insecta	Coleoptera	*Pseudanchomenusaptinoides* (Tarnier, 1860)	E	0	0	761	0	0	0	0
Insecta	Coleoptera	*Pseudechinosomanodosum* Hustache, 1936	E	0	0	16	0	0	0	0
Insecta	Coleoptera	Pseudophloeophagustenaxborgesi Stüben, 2022	E	106	130	464	0	460	2	3
Insecta	Coleoptera	*Pseudophloeophagustruncorum* (Stephens, 1831)	N	0	0	0	0	4	0	0
Insecta	Coleoptera	*Pseudoplectusperplexus* (Jacquelin du Val, 1854)	NA	0	1	0	0	1	0	0
Insecta	Coleoptera	*Psylliodeschrysocephalus* (Linnaeus, 1758)	I	1	0	1	0	0	1	0
Insecta	Coleoptera	*Psylliodesmarcida* (Illiger, 1807)	N	4	539	91	16	26	3	1
Insecta	Coleoptera	*Ptenidiumpusillum* (Gyllenhal, 1808)	I	3	1	2	0	16	10	0
Insecta	Coleoptera	*Pterostichusvernalis* (Panzer, 1796)	I	0	0	0	0	4	0	0
Insecta	Coleoptera	*Quediuscurtipennis* Bernhauer, 1908	NA	0	0	0	0	2	1	6
Insecta	Coleoptera	*Quediussimplicifrons* Fairmaire, 1862	NA	0	0	1	0	2	0	0
Insecta	Coleoptera	*Rhizophagusferrugineus* (Paykull, 1800)	I	0	0	1*	0	0	0	0
Insecta	Coleoptera	*Rhopalomesitestardyi* (Curtis, 1825)	I	4	0	12	0	2	0	0
Insecta	Coleoptera	*Rhyzobiuslophanthae* (Blaisdell, 1892)	I	0	0	0	0	6	0	0
Insecta	Coleoptera	*Rugilusorbiculatus* (Paykull, 1789)	NA	0	0	0	1	3	0	1
Insecta	Coleoptera	*Scymnusinterruptus* (Goeze, 1777)	N	0	1	1	0	64	0	0
Insecta	Coleoptera	*Scymnussuturalis* Thunberg, 1795	I	0	4*	0	0	0	0	0
Insecta	Coleoptera	*Sepedophiluslusitanicus* Hammond, 1973	NA	0	0	3	1	2	0	0
Insecta	Coleoptera	*Sericoderuslateralis* (Gyllenhal, 1827)	I	1	6	24	3	107	18	0
Insecta	Coleoptera	*Sirocalodesmixtus* (Mulsant & Rey, 1859)	I	0	0	0	0	1	0	0
Insecta	Coleoptera	*Sitonadiscoideus* Gyllenhal, 1834	I	3	1	2*	2	18	1	0
Insecta	Coleoptera	*Sphaeridiumbipustulatum* Fabricius, 1781	I	1	0	0	0	1	0	0
Insecta	Coleoptera	*Sphenophorusabbreviatus* (Fabricius, 1787)	I	4	0	0	0	19	0	0
Insecta	Coleoptera	*Stelidotageminata* (Say, 1825)	I	1	1	1	1	4	0	0
Insecta	Coleoptera	*Stenolophusteutonus* (Schrank, 1781)	N	0	0	0	0	4	0	0
Insecta	Coleoptera	*Stenomastaxmadeirae* Assing, 2003	NA	1	0	0	1*	2	0	0
Insecta	Coleoptera	*Stilbustestaceus* (Panzer, 1797)	N	0	2	1*	19	33	0	2
Insecta	Coleoptera	*Suniuspropinquus* (Brisout de Barneville, 1867)	NA	0	0	4	0	1	0	0
Insecta	Coleoptera	*Tachyporuschrysomelinus* (Linnaeus, 1758)	NA	54	16	18	146	26	13	13
Insecta	Coleoptera	*Tachyporusnitidulus* (Fabricius, 1781)	NA	14	30	27	83*	98	10	36
Insecta	Coleoptera	*Tarphiusfloresensis* Borges & Serrano, 2017	E	6	0	0	0	0	0	0
Insecta	Coleoptera	*Tarphiusfurtadoi* Borges & Serrano, 2017	E	0	0	11	0	0	0	0
Insecta	Coleoptera	*Tarphiusgabrielae* Borges & Serrano, 2017	E	0	0	2	0	0	0	0
Insecta	Coleoptera	*Tarphiusrufonodulosus* Israelson, 1984	E	0	0	0	0	0	0	1
Insecta	Coleoptera	*Trichiusarobustula* Casey, 1893	NA	0	0	0	1*	1	0	0
Insecta	Coleoptera	*Tychiuspicirostris* (Fabricius, 1787)	I	0	0	1	0	0	0	0
Insecta	Coleoptera	*Typhaeastercorea* (Linnaeus, 1758)	I	0	0	1	0	2	0	1
Insecta	Coleoptera	*Xantholinuslongiventris* Heer, 1839	NA	0	1	2	0	8	0	0
Insecta	Coleoptera	*Xyleborinusalni* Nijima, 1909	I	0	0	2	0	458	0	0
Insecta	Dermaptera	*Euborelliaannulipes* (Lucas, 1847)	I	20	36	0	29	1	84	0
Insecta	Dermaptera	*Forficulaauricularia* Linnaeus, 1758	I	7	112	18	91	0	16	18
Insecta	Dermaptera	*Labidurariparia* (Pallas, 1773)	N	0	0	0	1	0	2	0
Insecta	Ephemeroptera	*Cloeondipterum* (Linnaeus, 1761)	N	0	1	0	0	0	0	0
Insecta	Hemiptera	*Acalyptaparvula* (Fallén, 1807)	N	4	0	0	0	19	0	0
Insecta	Hemiptera	*Acizziauncatoides* (Ferris & Klyver, 1932)	I	2	0	386	1	34	0	0
Insecta	Hemiptera	*Acyrthosiphonloti* (Theobald, 1913)	N	0	0	0	0	1	0	0
Insecta	Hemiptera	*Anthocorisnemoralis* (Fabricius, 1794)	N	5	1	22*	0	3	1	0
Insecta	Hemiptera	*Aphrodeshamiltoni* Quartau & Borges, 2003	E	6	0	81	0	16	1	0
Insecta	Hemiptera	*Beosusmaritimus* (Scopoli, 1763)	N	0	0	0	0	1	0	0
Insecta	Hemiptera	*Brachystelesparvicornis* (A. Costa, 1847)	N	0	7*	0	0	3	0	0
Insecta	Hemiptera	*Buchananiellacontinua* (White, 1880)	I	0	1	23	0	1	0	1
Insecta	Hemiptera	*Campyloneuravirgula* (Herrich-Schaeffer, 1835)	N	4	28	317	200	33	0	0
Insecta	Hemiptera	*Cicadellaviridis* (Linnaeus, 1758)	I	0	0	0	0	3	0	0
Insecta	Hemiptera	***Cinarajuniperi* (De Geer, 1773)**	N	2177	277	2094	0	3008	8	0
Insecta	Hemiptera	*Cixiusazofloresi* Remane & Asche, 1979	E	972	0	0	0	0	0	0
Insecta	Hemiptera	*Cixiusazomariae* Remane & Asche, 1979	E	0	0	0	0	0	0	371
Insecta	Hemiptera	*Cixiusazopifajoazofa* Remane & Asche, 1979	E	0	151	0	0	0	0	0
Insecta	Hemiptera	***Cixiusazopifajoazopifajo* Remane & Asche, 1979**	E	0	0	7222	0	0	0	0
Insecta	Hemiptera	*Cixiusazoricusazoricus* Lindberg, 1954	E	0	0	0	0	3	0	0
Insecta	Hemiptera	*Cixiusazoricusazoropicoi* Remane & Asche, 1979	E	0	0	640	0	0	0	0
Insecta	Hemiptera	***Cixiusazoterceirae* Remane & Asche, 1979**	E	0	0	0	0	17922	0	0
Insecta	Hemiptera	*Cixiusinsularis* Lindberg, 1954	E	0	0	0	0	0	531	0
Insecta	Hemiptera	***Cyphopterumadcendens* (Herrich-Schäffer, 1835)**	N	1131	575	2671	1090	5497	31	5
Insecta	Hemiptera	*Emblethisdenticollis* Horváth, 1878	N	1	0	0	0	2	0	0
Insecta	Hemiptera	*Empicorisrubromaculatus* (Blackburn, 1889)	I	1	0	14	0	11	0	0
Insecta	Hemiptera	*Euphylluraolivina* (Costa, 1839)	I	0	0	0	1*	0	0	0
Insecta	Hemiptera	*Eupteryxazorica* Ribaut, 1941	E	94	89*	69	1	67	27	6*
Insecta	Hemiptera	*Eupteryxfilicum* (Newman, 1853)	N	34	12	21	4	7	0	0
Insecta	Hemiptera	*Euscelidiusvariegatus* (Kirschbaum, 1858)	N	0	0	12	0	3	0	0
Insecta	Hemiptera	*Geotomuspunctulatus* (A. Costa, 1847)	N	0	0	1*	0	2	0	0
Insecta	Hemiptera	*Heterotomaplanicornis* (Pallas, 1772)	N	0	11	7	5	1	4	0
Insecta	Hemiptera	*Kelisiaribauti* Wagner, 1938	N	3	0	5	1	377	1	5
Insecta	Hemiptera	***Kleidocerysericae* (Horváth, 1909)**	N	90	3909	195	2718	11260	1	1
Insecta	Hemiptera	*Lasiosomusenervis* (Herrich-Schäffer, 1835)	N	0	0	1*	0	0	0	0
Insecta	Hemiptera	*Loriculacoleoptrata* (Fallén, 1807)	N	10	0	323*	53*	248*	0	0
Insecta	Hemiptera	*Loriculaelegantula* (Bärensprung, 1858)	N	0	0	13	5	6*	0	0
Insecta	Hemiptera	*Lyctocoriscampestris* (Fabricius, 1794)	I	0	0	0	1	0	1	0
Insecta	Hemiptera	*Megamelodesquadrimaculatus* (Signoret, 1865)	N	0	1	316	0	18	6	0
Insecta	Hemiptera	*Microplaxplagiatus* (Fieber, 1837)	N	0	0	0	0	1*	0	0
Insecta	Hemiptera	*Miridiusquadrivirgatus* (A. Costa, 1853)	N	0	0	0	0	1*	0	0
Insecta	Hemiptera	*Monalocorisfilicis* (Linnaeus, 1758)	N	24	37	125	0	105	62	0
Insecta	Hemiptera	*Myzuscerasi* (Fabricius, 1775)	I	0	0	0	0	1	0	0
Insecta	Hemiptera	*Nabispseudoferusibericus* Remane, 1962	N	7	2	1	1	45	4	4
Insecta	Hemiptera	*Nezaraviridula* (Linnaeus, 1758)	I	0	0	1	0	0	0	0
Insecta	Hemiptera	*Nysiusatlantidum* Horváth, 1890	E	1	17	0	0	7	0	0
Insecta	Hemiptera	*Opsiusstactogalus* Fieber, 1866	N	0	0	1	0	0	0	0
Insecta	Hemiptera	*Oriuslaevigatuslaevigatus* (Fieber, 1860)	N	8	3	33	1	10	1	0
Insecta	Hemiptera	*Philaenusspumarius* (Linnaeus, 1758)	I	0	0	0	0	0	28	0
Insecta	Hemiptera	*Piezodoruslituratus* (Fabricius, 1794)	N	0	0	0	2*	27*	0	0
Insecta	Hemiptera	*Pilophorusconfusus* (Kirschbaum, 1856)	N	1	1	0	0	30	0	0
Insecta	Hemiptera	*Pilophorusperplexus* Douglas & Scott, 1875	N	0	0	0	0	9	0	0
Insecta	Hemiptera	***Pinalitusoromii* J. Ribes, 1992**	E	149	162	664	0	3095	48	167
Insecta	Hemiptera	*Plinthisusbrevipennis* (Latreille, 1807)	N	0	0	3	1	270	0	0
Insecta	Hemiptera	***Plinthisusminutissimus* Fieber, 1864**	N	0	16	3*	548*	9726	0	0
Insecta	Hemiptera	*Rhopalosiphoninuslatysiphon* (Davidson, 1912)	I	4	3	14	0	10	0	0
Insecta	Hemiptera	*Rhopalosiphumpadi* (Linnaeus, 1758)	I	0	0	1	0	1	0	0
Insecta	Hemiptera	*Saldulapalustris* (Douglas, 1874)	N	1	0	2	0	3	3	0
Insecta	Hemiptera	*Scolopostethusdecoratus* (Hahn, 1833)	N	8	119	6	7	80	0	14
Insecta	Hemiptera	*Siphantaacuta* (Walker, 1851)	I	0	0	163*	1*	2	0	0
Insecta	Hemiptera	*Strophingiaharteni* Hodkinson, 1981	E	37	242	186	77	2329	4	106
Insecta	Hemiptera	*Therioaphistrifolii* (Monell, 1882)	N	0	0	0	0	15	0	0
Insecta	Hemiptera	*Trigonotyluscaelestialium* (Kirkaldy, 1902)	N	0	0	0	0	1	0	0
Insecta	Hemiptera	*Triozalaurisilvae* Hodkinson, 1990	N	3	232	1134	0	1620	402	188
Insecta	Hymenoptera	*Hypoponeraeduardi* (Forel, 1894)	N	110	19	9	0	5	34	0
Insecta	Hymenoptera	*Lasiusgrandis* Forel, 1909	N	345	251	1595	212	906	7	5
Insecta	Hymenoptera	*Monomoriumcarbonarium* (Smith, 1858)	N	3	0	0	2	316	0	0
Insecta	Hymenoptera	*Tetramoriumcaespitum* (Linnaeus, 1758)	N	1	40	5	0	9	0	0
Insecta	Hymenoptera	*Tetramoriumcaldarium* (Roger, 1857)	I	0	0	1	1	82	0	0
Insecta	Neuroptera	*Chrysoperlalucasina* (Lacroix, 1912)	I	0	0	0	0	1	0	0
Insecta	Neuroptera	*Hemerobiusazoricus* Tjeder, 1948	E	69	26	92	0	904	26	193
Insecta	Orthoptera	*Eumodicogryllusbordigalensis* (Latreille, 1804)	I	0	0	3*	0	0	0	0
Insecta	Orthoptera	*Phaneropteranana* Fieber, 1853	N	0	0	1*	0	4	0	0
Insecta	Phasmida	*Carausiusmorosus* (Sinéty, 1901)	I	0	0	0	0	1	0	0
Insecta	Psocodea	*Atlantopsocusadustus* (Hagen, 1865)	N	15	8	25	26	532	6	0
Insecta	Psocodea	*Bertkauialucifuga* (Rambur, 1842)	N	15	32	99*	5*	233	0	0
Insecta	Psocodea	*Ectopsocusbriggsi* McLachlan, 1899	I	1246	36	424	79	303	242	343
Insecta	Psocodea	*Ectopsocusstrauchi* Enderlein, 1906	N	24	0	1	2	169	0	0
Insecta	Psocodea	*Elipsocusazoricus* Meinander, 1975	E	135	65	886	3	504	40	182
Insecta	Psocodea	*Elipsocusbrincki* Badonnel, 1963	E	470	147	290	9	1884	6	1
Insecta	Psocodea	*Trichadenotecnumcastum* Betz, 1983	I	0	0	1	8*	0	0	0
Insecta	Psocodea	*Trichopsocusclarus* (Banks, 1908)	N	171	133	2251	24	836	224	40
Insecta	Psocodea	*Valenzuelaburmeisteri* (Brauer, 1876)	N	0	62	103*	13*	981	45	5
Insecta	Psocodea	*Valenzuelaflavidus* (Stephens, 1836)	N	197	86	1299	315	1127	54	168
Insecta	Strepsiptera	*Elenchustenuicornis* (Kirby, 1815)	N	0	0	0	0	5	0	0
Insecta	Thysanoptera	*Aeolothripsericae* Bagnall, 1920	N	0	0	0	0	1	0	0
Insecta	Thysanoptera	*Aeolothripsgloriosus* Bagnall, 1914	N	0	0	4	16	204	0	0
Insecta	Thysanoptera	*Anisopilothripsvenustulus* (Priesner, 1923)	I	0	0	3	0	5	0	0
Insecta	Thysanoptera	*Aptinothripsrufus* (Haliday, 1836)	I	0	3	7	0	16	0	0
Insecta	Thysanoptera	*Ceratothripsericae* (Haliday, 1836)	N	1*	0	56	0	76	0	0
Insecta	Thysanoptera	*Heliothripshaemorrhoidalis* (Bouché, 1833)	I	30	216	36	31	773	1	0
Insecta	Thysanoptera	*Hercinothripsbicinctus* (Bagnall, 1919)	I	29	3	165	6	109	0	0
Insecta	Thysanoptera	*Hoplothripscorticis* (De Geer, 1773)	N	73	3	217	1	183	4	6
Insecta	Trichoptera	*Limnephilusatlanticus* Nybom, 1948	E	23	18	67	0	7	61	0
Symphyla	Symphyla	*Scutigerellaimmaculatus* (Newport, 1845)	I	0	0	0	0	3	0	0

**Table 3.** List of orders of arthropods mentioning the number of species or subspecies identified, as well as the number of new records for each Island (FLO-Flores; FAI – Faial; GRA – Graciosa; PIC – Pico; TER – Terceira; SMG – São Miguel; SMR – Santa Maria). Data for Araneae from Pico and Terceira Islands are not mentioned because these are already available in Costa and Borges (2021) and Lhoumeau et al. (2022).

**Class**	**Order**	**FLO**	**FAI**	**GRA**	**PIC**	**TER**	**SMG**	**SMR**
Arachnida	Araneae	42	**2 NEW** 37	**5 NEW** 21	---	---	**1 NEW** 27	**1 NEW** 18
Arachnida	Opiliones	2	2	1	2	2	2	
Arachnida	Pseudoscorpiones	1	1	2	3	3		
Chilopoda	Geophilomorpha	1			**1 NEW** 2	1	1	
Chilopoda	Lithobiomorpha	1	1		1	1		
Chilopoda	Scolopendromorpha					1	1	
Chilopoda	Scutigeromorpha	1		1	1	1		
Diplopoda	Chordeumatida				1	1	1	
Diplopoda	Julida	7	5	3	**4 NEW** 7	6	5	5
Diplopoda	Polydesmida	2	1	1	3	2		
Insecta	Archaeognatha	1	1	1	2	2		
Insecta	Blattodea	1	1		1	1	1	1
Insecta	Coleoptera	**1 NEW** 57	**4 NEW** 46	**11 NEW** 45	**8 NEW** 88	**3 NEW** 108	**2 NEW** 49	**2 NEW** 45
Insecta	Dermaptera	2	2	3	1	1	3	1
Insecta	Ephemeroptera		1					
Insecta	Hemiptera	27	**2 NEW** 26	**5 NEW** 23	**6 NEW** 39	**5 NEW** 50	25	**1 NEW** 19
Insecta	Hymenoptera	4	3	3	4	6	2	1
Insecta	Neuroptera	1	1		1	2	1	1
Insecta	Orthoptera		1		**2 NEW** 2	2	2	2
Insecta	Phasmida					1		
Insecta	Psocodea	9	8	**3 NEW** 10	**2 NEW** 11	10	9	7
Insecta	Strepsiptera					1		
Insecta	Thysanoptera	**1 NEW** 4	5	4	7	8	3	2
Insecta	Trichoptera	1	1		1	1	1	
Symphyla	Symphyla					1		
TOTAL		**2 NEW** 164	**8 NEW** 143	**24 NEW** 118	**23 NEW** 177	**8 NEW** 212	**3 NEW** 133	**4 NEW** 102
